# Practical high-performance lead-free piezoelectrics: structural flexibility beyond utilizing multiphase coexistence

**DOI:** 10.1093/nsr/nwz167

**Published:** 2019-11-05

**Authors:** Qing Liu, Yichi Zhang, Jing Gao, Zhen Zhou, Dong Yang, Kai-Yang Lee, Andrew Studer, Manuel Hinterstein, Ke Wang, Xiaowen Zhang, Longtu Li, Jing-Feng Li

**Affiliations:** 1 State Key Laboratory of New Ceramics and Fine Processing, School of Materials Science and Engineering, Tsinghua University, Beijing 100084, China; 2 Institute for Applied Materials, Karlsruhe Institute of Technology, Karlsruhe 76131, Germany; 3 Australian Centre for Neutron Scattering, Australian Nuclear Science and Technology Organisation, Lucas Heights, NSW 2234, Australia

**Keywords:** piezoelectricity, lead-free, potassium–sodium niobite, structural flexibility, temperature stability

## Abstract

Due to growing concern for the environment and human health, searching for high-performance lead-free piezoceramics has been a hot topic of scientific and industrial research. Despite the significant progress achieved toward enhancing piezoelectricity, further efforts should be devoted to the synergistic improvement of piezoelectricity and its thermal stability. This study provides new insight into these topics. A new KNN-based lead-free ceramic material is presented, which features a large piezoelectric coefficient (*d*_33_) exceeding 500 pC/N and a high Curie temperature (*T*_c_) of  ∼200°C. The superior piezoelectric response strongly relies on the increased composition-induced structural flexibility due to lattice softening and decreased unit cell distortion. In contrast to piezoelectricity anomalies induced via polymorphic transition, this piezoelectricity enhancement is effective within a broad temperature range rather than a specific small range. In particular, a hierarchical domain architecture composed of nano-sized domains along the submicron domains was detected in this material system, which further contributes to the high piezoelectricity.

## INTRODUCTION

Piezoelectric materials are a unique medium for the conversion between mechanical and electrical energy and play a vital role in a variety of applications such as sensors, transducers, and actuators [1,2]. For decades, the global piezoelectric materials market was monopolized by lead zirconium titanate (PZT) based materials. However, a strong increase in environmental concerns has driven tremendous efforts toward lead-free substitutes [2–4]. Among the various types of lead-free substitutes, their overall excellent performance highlights (Na,K)NbO_3_ (KNN)-based ceramics as one of the most promising candidates [3,5–7]. The breakthrough by Saito *et al*. has manifested the promising potential of KNN-based ceramics [5]. Over the last decade, developing KNN-based ceramics with large piezoelectricity has become a hot research topic in both academic and industrial fields. Extensive studies have concentrated on constructing phase transitions near room temperature [5,8–11]. Forming a rhombohedral–tetragonal R-T (or rhombohedral–orthorhombic–tetragonal, R-O-T) phase boundary has been considered to be an effective strategy to achieve an ultrahigh *d*_33_ value in KNN-based ceramics [4,9–12]. However, not all KNN-based piezoceramics with the R-T (or R-O-T) phase boundary exhibit such a large *d*_33_ [13–16]. Although constructing a R-T (or R-O-T) phase boundary is not difficult as long as appropriate dopants are utilized, obtaining high piezoelectricity via this method still remains very challenging [4,9–11]. Furthermore, the origins of superior piezoelectricity in lead-free ceramics remain controversial, thus obstructing the further development of lead-free ceramics.

Sufficient temperature stability of the piezoelectricity is another essential requirement for practical applications. Several novel approaches, such as inducing electrically enhanced diffused polymorphic phase transition (EED-PPT) and introducing diffused R-O-T phase transition, have been proposed to develop temperature-insensitive lead-free piezoelectric materials [14,17–19]. However, it should be noted that the temperature-insensitive piezoelectricity originating from the diffused polymorphic phase transition is essentially induced by the degraded singularity of polymorphic phase transition (PPT) effects, which comes at the expense of high piezoelectricity [19]. An ideal scenario to achieve high and thermally stable piezoelectricity would be that the property enhancement is only related to the composition-induced free-energy instability [20]. Recent studies have provided evidence that the ultrahigh piezoelectricity of relaxor systems could be the result of structural instability associated with an interaction competition between the static structure of bulk and composition-induced local heterogeneities, i.e. local polar clusters known as polar nanoregions (PNRs) [20–25]. A further typical example of practical interest is the morphotropic phase boundary (MPB), in the vicinity of which high piezoelectric performance might arise from the anomalous softening of dielectric susceptibility and elastic moduli, resulting from composition-induced structural instability [20,26–29]. Therefore, both local structural instability and structure softening by chemical modification could contribute to temperature-independent property enhancement. This should be further emphasized to achieve high and thermally stable piezoelectricity. Consequently, the current study has developed a new lead-free ceramic composition: 0.93(Li*_x_*Na_0.52_K_0.48-*x*_)(Nb_1-*y*_,Sb*_y_*)O_3_-0.05BaZrO_3_-0.02(Bi_0.5_,Na_0.5_)HfO_3_ with 1 wt% MnO_2_ as a sintering aid (abbreviated as L*_x_*KNNS*_y_*-5BZ-2BNH-1Mn). The impacts of Li and Sb contents on the piezoelectric response and its thermal stability were investigated, the results of which clarified the piezoelectricity enhancement mechanisms. Enhanced thermal stability of piezoelectricity was observed in the optimum compositions with a large *d*_33_ exceeding 500 pC/N and a high *T*_c_ of  ∼200°C. This is strongly associated with the increased composition-induced structural flexibility, which benefits from reduced unit cell distortion and lattice softening.

## RESULTS AND DISCUSSION

**Figure 1.**
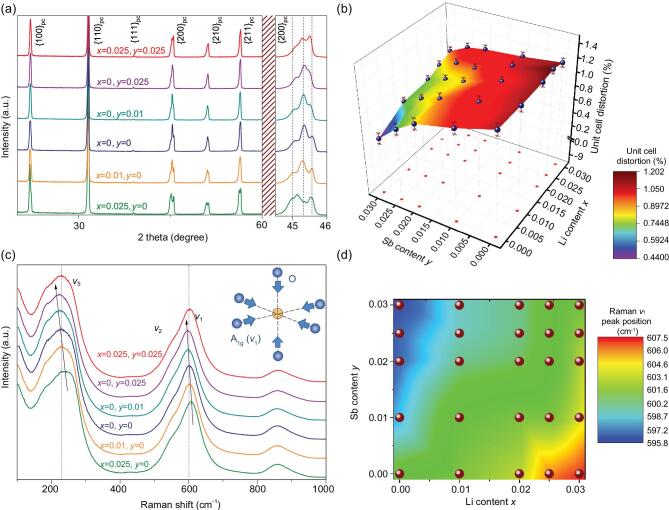
(a) XRD patterns for representative L*_x_*KNNS*_y_*-5BZ-2BNZ-1Mn samples. (b) Unit cell distortion (calculated by (c/a – 1) × 100%) of the global lattice for L*_x_*KNNS*_y_*-5BZ-2BNZ-1Mn samples. (c) Room-temperature Raman spectra of representative L*_x_*KNNS*_y_*-5BZ-2BNH-1Mn samples. The inset shows a schematic illustration of the breath-type stretching A_1g_ mode. (d) Raman *v*_1_ peak position of the L*_x_*KNNS*_y_*-5BZ-2BNH-1Mn samples.

The L*_x_*KNNS*_y_*-5BZ-2BNZ-1Mn ceramics exhibited a pure perovskite structure, and no evidence of macroscopic impurity could be found. This indicates that BZ and BNH had diffused into the L_x_KNNS_y_ lattices to form a stable solid solution (see Fig. S1, Supporting Information). As shown in Fig. 1a, these ceramics all featured multiphase coexistence according to the apparent splitting of 002_pc_ reflection peaks around 2θ ≈ 45° [3,4,10,11,13–15,19,30–32]. With increasing Sb content, the feature of the R phase became more prominent in the 002_pc_ reflection peaks. With increasing Li content, the 002_pc_ reflection peaks of the ceramics exhibited more characteristics of the T and O coexistent phases. Based on the 2θ interval between the leftmost 002_pc_ peak and the rightmost 002_pc_ peak, as a function of compositional change, the unit cell distortion (calculated by (c/a – 1) × 100%) of the global lattice decreased with increasing Sb content. Furthermore, it showed a mild increasing trend with increasing concentration of Li as shown in Fig. 1b. The increased lattice distortion can be interpreted as a lowering of symmetry, which is believed to be associated with octahedral tilting [33,34]. The need for this octahedral tilting is determined by the volume matching degree between the BO_6_ octahedron and the AO_12_ polyhedron. This can be evaluated according to the tolerance factor *t* (determined by (*R*_A_ + *R*_O_)/[$\sqrt{2}$(*R*_B_ + *R*_O_)], where *R*_A_, *R*_B_, and *R*_O_ are the radii of A, B, and O ions, respectively) [33]. The increased tolerance factor from a value below 1 indicates a better matching between the BO_6_ octahedron and the AO_12_ polyhedron as well as the reduced potential of octahedral tilting to accommodate cations. The tolerance factor increases with the B-site cation substitution by Sb^5+^ but decreases with the A-site substitution by Li^+^ due to the smaller radii of Sb^5+^ and Li^+^ compared with those of Nb^5+^ and K^+^, respectively [35]. Thus, Li doping can increase the potential of octahedral tilting while the opposite effects are expected in Sb doping, which might account for the changing trend of the lattice distortion.

Changes of short-range structures can be revealed by the collected Raman spectra (see Fig. S2). As shown in Fig. 1c, three broad bands were identified in all these ceramics, indicating the highly disordered lattice matrix [19]. As indicated in the inset of Fig. 1c, the A_1g_ mode denotes the breath-type stretching of the oxygen octahedron, which is naturally influenced by A–O or B–O bonds. Thus, the influence of Li and Sb contents on the perovskite structure can be indirectly detected by investigating the change of the A_1g_ mode. The A_1g_ mode wavenumber showed a decreasing trend with increasing Sb content but an increasing trend with increasing Li content, as shown in Fig. 1d. The reduced wavenumber could be attributed to the weakening of the bonding strength, which in turn is associated with the lower force constant [19,36]. The above changing trend is expected since the Sb–O bond energy is much lower than the Nb–O bond energy, while the Li–O bond energy is higher than the K–O bond energy [37,38]. Given the lower force constant and the less tight octahedral environment due to the smaller radius of Sb^5+^ compared with Nb^5+^, the matrix was considered to be softened by the introduction of Sb. More covalent KNN-based perovskite also occurred due to the much higher electronegativity of the Sb^5+^ compared with Nb^5+^. Collectively, these factors make it easier for ferroelectric active B-site cations to move between equivalent off-centering positions in the oxygen octahedral. This can result in a lower energy barrier between ferroelectric states [34].

When investigating the temperature dependences of the permittivity for the L*_x_*KNNS*_y_*-5BZ-2BNZ-Mn unpoled samples (see Fig. S3), a relaxed bump situated near room temperature signifying a PPT was observed in the ϵ_r_–*T* curves of all samples.

**Figure 2.**
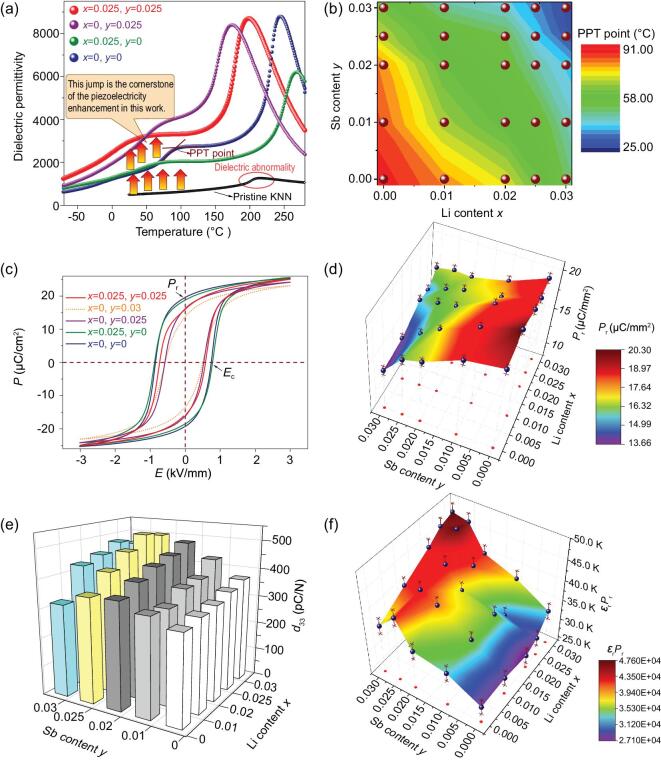
(a) Temperature dependence of the dielectric permittivity for representative L*_x_*KNNS*_y_*-5BZ-2BNZ-1Mn samples. (b) The compositional dependence of the PPT point for L*_x_*KNNS*_y_*-5BZ-2BNH-1Mn samples. (c) Room-temperature *P–E* loops of the representative L*_x_*KNNS*_y_*-5BZ-2BNZ-1Mn samples. (d) *P*_r_ extracted from the room-temperature *P*–*E* loops of the L*_x_*KNNS*_y_*-5BZ-2BNZ-1Mn samples. (e) The room-temperature piezoelectric coefficient *d*_33_ of the L*_x_*KNNS*_y_*-BZ-BNH-1Mn samples. (f) ϵ_r_·*P*_r_ of the L*_x_*KNNS*_y_*-5BZ-2BNZ-1Mn samples.

As shown in Fig. 2a, the ϵ_r_ level in the temperature range above the PPT point showed an increasing tendency as Sb content increased, while the opposite effects were observed for Li. Figure 2b shows the PPT points of the L*_x_*KNNS*_y_*-5BZ-2BNZ-Mn samples, which were extracted according to the method illustrated in Fig. 2a. The PPT point showed a decreasing trend with increasing Li or Sb contents. Similar phase transition points were observed in the samples incorporated with a similar sum of Li and Sb contents.

Piezoelectricity is closely related to not only ferroelectricity but also dielectricity. *P*–*E* loops were measured to investigate the impacts of Li and Sb contents on the ferroelectricity of the L*_x_*KNNS*_y_*-5BZ-2BNZ-1Mn samples (see Fig. S4). As shown in Fig. 2c, the *P*–*E* loop changed to be slimmer as Sb content increased, while the opposite trend occurred as Li content increased. Clearly, increasing the Sb content induced a lower *E*_c_ while increasing Li content resulted in a higher *E*_c_. The compositional dependence of the remanent polarization *P*_r_ extracted from the *P*–*E* loops is summarized in Fig. 2d. Significant reduction of *P*_r_ was observed when the Sb content was increased. The decreased unit cell distortion can degrade the spontaneous polarization, which then leads to a reduction of the macroscopic remanent polarization. It should be noted that increasing the Li content was found to help maintain a relatively larger remanent polarization in the ceramic samples modified with high Sb content.

**Figure 3.**
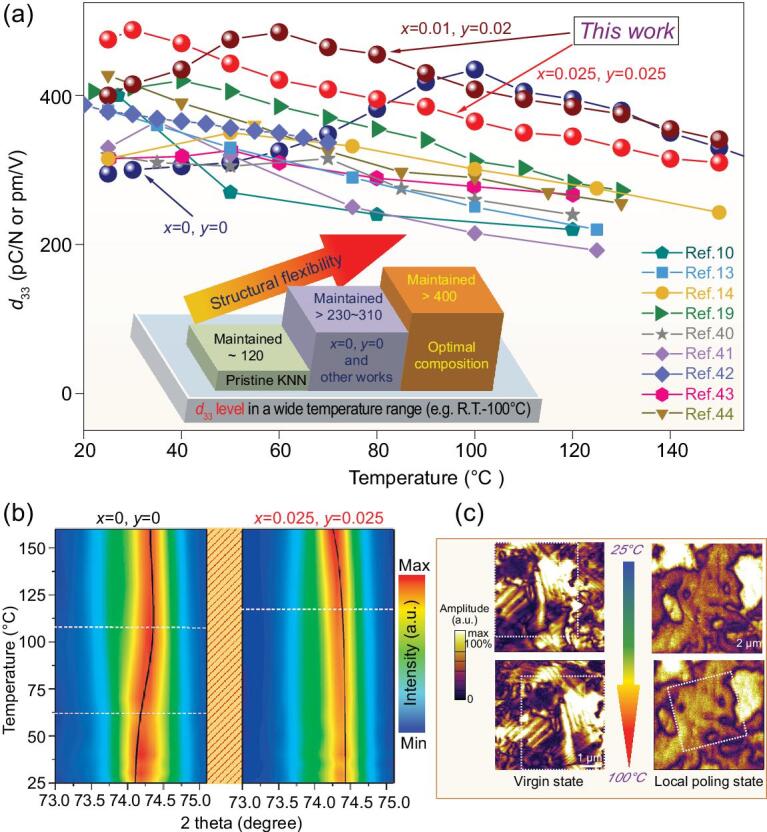
(a) Comparison of the temperature dependences of *d*_33_ for various representative KNN-based ceramics. (b) Temperature-dependent neutron diffraction intensity mapping of the 002_pc_ reflection of the *x* = 0, *y* = 0 and *x* = 0.025, *y* = 0.025 samples. (c) Out-of-plane PFM amplitudes of the *x* = 0.025, *y* = 0.025 sample in the virgin state and local poling state at 25°C and 100°C.

Figure 2e shows the room-temperature piezoelectric coefficient *d*_33_ of L*_x_*KNNS*_y_*-BZ-BNH-1Mn ceramics. In general, Sb-modified samples exhibited a larger *d*_33_ than Li-modified samples. *d*_33_ increased as Li or Sb contents increased but extensive addition of Sb or Li deteriorated *d*_33_. A similar trend could also be observed with regard to the planar electromechanical coupling factor *k*_p_ (see Fig. S5). A peak *d*_33_ value ∼510 pC/N was attained in the *x* = 0.025, *y* = 0.025 composition. This *d*_33_ ranks among the largest *d*_33_ values reported in current KNN-based ceramics [4,9–11]. The relationship among the piezoelectric coefficient *d*_33_, dielectric permittivity ϵ_r_, and remanent polarization *P*_r_ has been roughly described as *d*_33_ ∝ ϵ_r_·*P*_r_ in the literature [4,9–11,19]. The relationship *d*_33_ ∝ ϵ_r_·*P*_r_ was also validated in this work, as shown in Fig. 2f. The optimal ϵ_r_·*P_r_* was achieved in the composition around *x* = 0.025, *y* = 0.025, consistent with the largest *d*_33_. As mentioned above, introducing Li and Sb into the KNN matrix did not enhance *P*_r_. The enhancement of ϵ_r_·*P*_r_ was mainly due to the significant enhancement of ϵ_r_, the origin of which could be attributed to the following two mechanisms. One is the dielectric abnormality adjusted by shifting the PPT point close to room temperature by further introducing both Li and Sb. The other is the dielectricity enhancement in the wide temperature range (referred to as dielectricity ‘jumping’), which can be clearly observed when increasing the Sb content (see Fig. S5). The dielectricity ‘jumping’, induced by the various dopants, such as BZ-BNH and Sb, constitutes the main source of the dielectricity enhancement when compared with that of the pristine KNN. This is deemed the cornerstone of the piezoelectricity enhancement in this work.

Investigating the thermal stability of the piezoelectric performance can help to unravel the origin of the property enhancement and also provides valuable information for practical applications. Our previous work showed that the thermal stability of the piezoelectric response could be estimated by measuring field-dependent piezoelectric coefficient *d*_33_(*E*) curves at different temperatures [10,39]. According to this method, the *in situ* thermal stability of the *d*_33_ of the representative compositions was evaluated, and the results are shown in Figs 3a and S6. As expected, all compositions exhibited the highest *d*_33_ around their phase transition temperatures, and a monotonically decreasing trend was observed when the temperature deviated from the phase transition point. These results were consistent with the temperature dependence of the ϵ_r_·*P*_r_–*T* curves (see Fig. S7). It is worth noting that the present optimal materials in this study demonstrate higher *d*_33_ values and better temperature stability when compared with recently reported KNN-based ceramics, as shown in Fig. 3a [10,13,14,18,19,40–44]. Piezoelectricity enhancement can be witnessed in a broad temperature range (also see Fig. S8). Since piezoelectric abnormality induced by PPT is naturally limited to a specific and small temperature range, the PPT effect is not considered as the main piezoelectricity enhancement mechanism in this study. Instead, we propose that composition-induced structural flexibility, the features of which will be described below, is the main contributing factor for the high piezoelectricity and the dielectricity ‘jumping’. Comparing the results of the temperature dependence of *d*_33_, composition-induced structural flexibility accounts for a more than 300% piezoelectricity increase in contrast to that of pristine KNN.

Thermal stability is significantly influenced by the phase structure evolution; thus, temperature-dependent neutron diffraction (ND) measurements on two selected samples, *x* = *y* = 0 and *x* = *y* = 0.025, were conducted. Figure 3b shows the 002_pc_ reflection of the ND patterns at a temperature range of 20–160°C. The evolution of the 002_pc_ reflections with temperature increasing for the *x* = *y* = 0 sample was more obvious than that of the *x* = *y* = 0.025 sample. The latter almost stood still in the temperature range between approximately 20°C and 100°C. The divided three different parts of the temperature-dependent 002_pc_ reflections for the *x* = *y* = 0 corresponded to the temperature dependence of the *d*_33_ (*E* = 0) and ϵ_r_. These were associated with two sudden temperature-induced structural changes. In contrast, the phase structure of the *x* = *y* = 0.025 sample experienced a gradual change over a broad temperature range. The diffused thermally stable structure might be partially responsible for the high thermal stability of the piezoelectric performance.

The piezoelectric property is also intimately associated with the domain morphology. The micro-scale domain morphology was investigated via piezoresponse force microscopy (PFM). *In situ* observations of the domain morphologies of the virgin state and the local poling state for the *x* = *y* = 0.025 sample were investigated at different temperatures. Figure 3c shows the amplified domain morphologies, where no significant change was observed as the temperature increased from 25°C to 100°C (full data can be found in Fig. S9). The thermally stable micro-scale domain structure might also contribute to the noteworthy thermal stability of *d*_33_.

**Figure 4.**
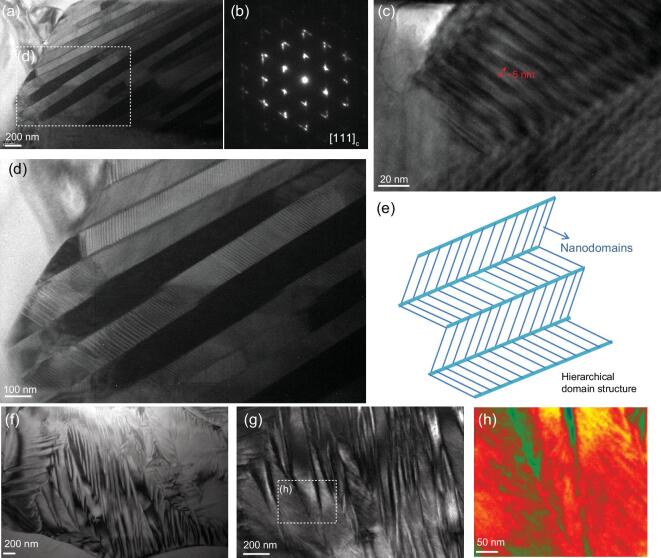
(a, c, d) Bright-field TEM images of the *x* = 0.025, *y* = 0.025 sample, showing hierarchical domain configuration and the corresponding selected area electron diffraction (SAED) pattern (b). (e) Schematic diagram of the hierarchical nanodomain architecture. (f–h) Bright-field TEM images of the *x* = 0.025, *y* = 0.025 sample showing strip-like and fibrous nanodomain patterns.

The underlying mechanisms of high piezoelectricity of the *x* = *y* = 0.025 ceramics were further explored via transmission electron microscopy (TEM). Substructural twinning was observed at the nanoscale, as shown in Fig. 4a, c, d. Stripe sub-micron domains were well arranged and composed by lamellar nanodomains, and both domains exhibited strict alternation. The average width of the lamellar nanodomains was approximately 5–10 nm, which is much smaller than nanotwinned structures that have been extensively reported in other lead-based and lead-free materials [4,10,11,14,19,45–51]. As shown in Fig. 4b, the corresponding electron diffraction pattern demonstrated streaking and elongating of the reflection spots, which was attributed to the slim nanodomains [46]. Clearly, the current material possessed a hierarchical nanodomain architecture as outlined in Fig. 4e [49]. It is worth noting that the domain configuration is a 3D architecture; however, the domain morphology observed using TEM only shows a 2D projection. This can be influenced by various factors such as the viewing direction, the thickness of the specimen, and boundary conditions [47,48,51]. It is common to observe utterly different morphologies in the TEM investigations; however, useful structural information can still be verified. Domain patterns featured with irregular fringe contrast or fibrous structures were observed as illustrated in Figs 4f–h and S10. Interestingly, traces of numerous nanodomains within sub-micron domains could be found in these domain patterns, which also possess a hierarchical characteristic. Recently, it has been reported that the fragmentation of local structure can contribute to the enhancement of the piezoelectric response [24,25]. An *in situ* TEM investigation of the electric-field-driven evolution of the domain structure showed that the existence of nanodomains is closely related to the extrinsic piezoelectric effect. This is because the real-time response occurred in nanodomains rather than the visibly unchanged micro-domain structures [47]. The facilitation of polarization reorientation under external stimulation enabled the miniaturization of the domain structure due to the drastic decrease of the domain wall energy [20,46,50]. The transformation stress between two polarization states of different phases can also be alleviated by the nanotwinned structure, which induces lattice softening [46,47,52]. Consequently, a hierarchical domain configuration that consists of nanodomains and nanotwins can lead to both a nearly vanishing polarization anisotropy and elastic softening. This ultimately results in the enhancement of the piezoelectric response [53].

**Figure 5.**
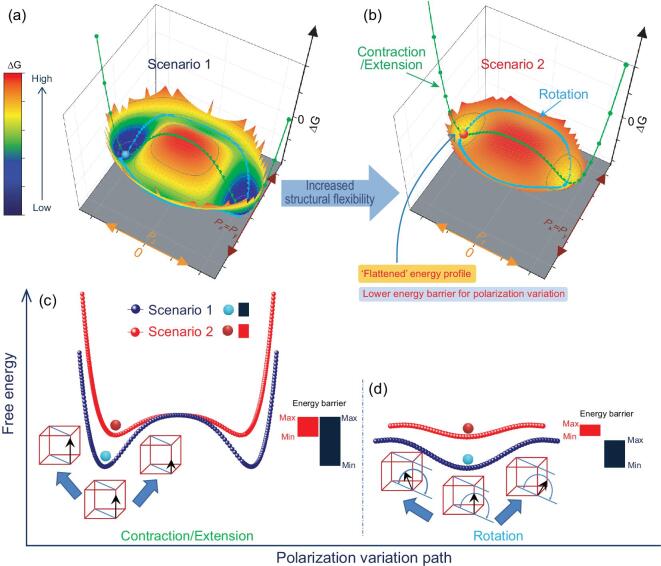
Phenomenological illustration of the piezoelectricity enhancement associated with increased structural flexibility. (a, b) Comparison of the energy landscape with polarization variation under the condition of *P_x_* = *P_y_* between two ferroelectric systems before and after additional structural flexibility is introduced. (c) Comparison of the energy landscapes for the polarization contraction/extension paths of scenario 1 and scenario 2 according to the 3D surfaces in (a) and (b), respectively. (d) Comparison of the energy landscapes for the polarization rotation paths of scenario 1 and scenario 2 according to the 3D surface in (a) and (b), respectively.

Based on these investigations, a phenomenological analysis of the piezoelectricity enhancement mechanism was conducted. In this study, the unit cell distortion of the global lattice decreased with increasing Sb content and increased with increasing Li content. The reduction of the unit cell distortion indicates a decrease in crystalline anisotropy, which can result in a smaller free energy barrier among the ferroelectric/paraelectric phases. Furthermore, the presence of lattice softening can alleviate transformation stress during lattice deformation. This favors a lower energy barrier during the polarization variation between ferroelectric states. Structural flexibility can arise from all these factors, leading to an ease of polarization variation and thus ‘soft’ behavior, expressed as low *E*_c_ and high dielectricity (see Fig. S4). However, the decreased unit cell distortion can also exert negative effects. It can decrease the spontaneous polarization, which then leads to the reduction of the macroscopic remanent polarization. The lowered polarization value might counteract the positive effect of the easier polarization variation when the unit cell distortion becomes too small. Thus, the lower unit cell distortion ratio might contribute to the piezoelectric response; however, an optimum value exists. The obtained experimental results indicate that introducing both Sb and Li elements into the ceramics was verified to be an effective way to achieve both the ‘soft’ behavior and maintaining the macroscopic remanent polarization. Possible energy landscapes can be generated by the suitable arrangement of parameters, while fully considering the above factors in the framework of the Landau–Ginzburg–Devonshire model [20,54]. This might help to understand the property enhancement due to increased structural flexibility. Figure 5 depicts the dissimilarity of energy landscapes for one polarization variation path between the following two scenarios: with and without increased structural flexibility. For the consistence of experimental results in this study, the tetragonal phase is considered as the initial and most stable phase in both cases. To aid an intuitive understanding, the energy landscapes are presented in a colorful 3D surface, as shown in Fig. 5a and b. This exhibits the change of free energy via the polarization variation path under the condition of polarization components *P_x_* = *P_y_*. Moreover, the energy landscapes of two typical polarization variation paths, namely, constriction/extension and rotation [55,56], are extracted from the 3D surfaces and compared, as shown in Fig. 5c and d, respectively. Decreased anisotropy of the free energy with polarization can be perceived when the energy barrier between various ferroelectric/paraelectric states decreases. A more ‘flattened’ energy profile can be established by cautiously increasing the composition-induced structural flexibility, which can result from lattice softening [46,53] and reduced unit cell distortion. The induced ‘flattened’ energy profile contributes to the facilitation of the polarization variation, thus accounting for the piezoelectricity enhancement of Li and Sb co-modified ceramics.

## CONCLUSION

In summary, enhanced piezoelectricity with improved thermal stability was achieved in L*_x_*KNNS*_y_*-BZ-BNH-1Mn ceramics, and its physical origins were systematically studied via comparative analyses. The softening effect and the optimum unit cell distortion are indispensable for large *d*_33_ exceeding 500 pC/N, achieved in this work. The presence of a hierarchical domain structure played a vital role in synergistically achieving reduced polarization anisotropy and elastic softening. This results in enhancements in the piezoelectric properties and thermal stability. We believe that this work can pave the way for the exploration of high-performance piezoceramics with excellent thermal reliability.

## METHODS

### Sample preparation

Lead-free ceramic samples of the nominal composition 0.93(Li*_x_*Na_0.52_K_0.48-*x*_)(Nb_1-*y*_,Sb*_y_*)O_3_-0.05BaZrO_3_-0.02(Bi_0.5_,Na_0.5_)HfO_3_ with 1 wt% MnO_2_ as a sintering aid [39] (abbreviated as L*_x_*KNNS*_y_*-5BZ-2BNH-1Mn, 0 *≤ x ≤* 0.03, 0 *≤ y ≤* 0.03) were synthesized via conventional ceramic processing. Firstly, the precursor oxides powders, including Li_2_CO_3_ (99%), Na_2_CO_3_ (99.8%), K_2_CO_3_ (99%), Nb_2_O_5_ (99.99%), Sb_2_O_3_ (99.99%), BaCO_3_ (99.95%), ZrO_2_ (99.9%), HfO_2_ (99.99%), and Bi_2_O_3_ (99.99%), were weighed according to their stoichiometric ratio and were then homogeneously mixed in ethanol using a planetary ball mill for 24 h. Calcination of the dried mixture was performed at 950°C for 4 h, and then the resultant powders were subjected to ball milling again with 1.0 wt% MnO_2_ as a sintering aid for 24 h. After drying, the powder mixtures were pressed into compacted disks of 10 mm in diameter, which was followed by cold isostatic pressing at 200 MPa for 2 min. The green pellets were sintered in the temperature range between 1080°C and 1180°C for 6 h.

### Crystal structure and microstructure analysis

The crystal structure was determined by an X-ray diffractometer (XRD, D/Max 2500; Rigaku, Tokyo, Japan) with a Cu *K*_α1_ (λ = 1.5405 Å) monochromator. The Raman spectra were collected by a Raman spectrophotometer (LabRAM HR, HoRIBA Jobin Yvon, France) with a 633 nm laser. Piezoresponse force microscope (PFM) observations were conducted using a commercial atomic force microscope (MFP-3D, Asylum Research, USA) with the functionality of a PFM. Additionally, to obtain TEM specimen, the as-sintered disks were first mechanically polished to around 20 μm in thickness. Lamellar samples were further reduced to reach electron transparency by using argon-ion beam milling (Gatan PIPS 695, Gatan Inc., USA) with an acceleration voltage of 0.1–6 kV. A high-resolution TEM (JEOL 2100, JEOL, Japan), which operated at 200 kV, was used to conduct TEM investigations. Neutron diffraction measurements were conducted using a high-intensity powder diffractometer (Wombat) at the Australian Nuclear Science and Technology Organisation (ANSTO). A CaAlNaF_3_ standard sample was used to determine the wavelength of the neutron beam, which was refined to 2.41962(6) Å. The dimension of the samples was 3.5 × 3.5 ×30 mm [3].

### Electrical property measurements

The as-sintered pellets were first ground to 1 mm thickness. Two surfaces of the samples, which were polished by using silicon carbide papers, were painted with silver pastes burnt in afterwards at 600°C for 30 min. The measurement of the temperature dependence of permittivity was conducted under 1 kHz during the heating process (2°C/min) using a precision LCR meter (TH2827C, Changzhou Tonghui Electronic Co., China) with a temperature-regulated chamber. The quasistatic piezoelectric coefficient *d*_33_ was measured by a Berlincourt meter (ZJ-3A, Institute of Acoustics, Chinese Academy of Sciences, China). Other ferroelectric and piezoelectric parameters including the piezoelectric coefficient *d*_33_(*E*), the unipolar strain *S*(*E*), and polarization *P*(*E*) hysteresis loops were measured by the same apparatus and method used previously [19,57].

## Supplementary Material

nwz167_Supplemental_File

## References

[1] Jaffe B , Cook WR, Jaffe H. Piezoelectric Ceramics. London: Academic, 1971.

[2] Rödel J , Li JF. Lead-free piezoceramics: status and perspectives. MRS Bull 2018; 43: 576–80.

[3] Wu J , Xiao D, Zhu J. Potassium-sodium niobate lead-free piezoelectric materials: past, present, and future of phase boundaries. Chem Rev 2015; 115: 2559–95. 25792114 10.1021/cr5006809

[4] Wu B , Wu H, Wu J et al. Giant piezoelectricity and high Curie temperature in nanostructured alkali niobate lead-free piezoceramics through phase coexistence. J Am Chem Soc 2016; 138: 15459–64. 27933925 10.1021/jacs.6b09024

[5] Saito Y , Takao H, Tani T et al. Lead-free piezoceramics. Nature 2004; 432: 84–7. 15516921 10.1038/nature03028

[6] Mgbemere HE , Hinterstein M, Schneider GA. Investigation of the structure and electrical properties of (K_x_Na_0.96-x_Li_0.04_)(Nb_0.96-y_Ta_y_Sb_0.04_)O_3_ piezoelectric ceramics modified with manganese. J Am Ceram Soc 2013; 96: 201–8.

[7] Mgbemere HE , Hinterstein M, Schneider GA. Structural phase transitions and electrical properties of (K_x_Na_1-x_)NbO_3_-based ceramics modified with Mn. J Eur Ceram Soc 2012; 32: 4341–52.

[8] Li JF , Wang K, Zhu FY et al. (K, Na) NbO_3_-based lead-free piezoceramics: fundamental aspects, processing technologies, and remaining challenges. J Am Ceram Soc 2013; 96: 3677–96.

[9] Wang X , Wu J, Xiao D et al. Giant piezoelectricity in potassium-sodium niobate lead-free ceramics. J Am Chem Soc 2014; 136: 2905–10. 24499419 10.1021/ja500076h

[10] Zheng T , Yuan Y, Lv X et al. Structural origin of enhanced piezoelectric performance and stability in lead free ceramics. Energy Environ Sci 2017; 10: 528–37.

[11] Xu K , Li J, Lv X et al. Superior piezoelectric properties in potassium-sodium niobate lead-free ceramics. Adv Mater 2016; 28: 8519–23. 27441456 10.1002/adma.201601859

[12] Zuo R , Fu J. Rhombohedral–tetragonal phase coexistence and piezoelectric properties of (NaK)(NbSb)O_3_-LiTaO_3_-BaZrO_3_ lead-free ceramics. J Am Ceram Soc 2011; 94: 1467–70.

[13] Wang R , Wang K, Yao F et al. Temperature stability of lead-free niobate piezoceramics with engineered morphotropic phase boundary. J Am Ceram Soc 2015; 98: 2177–82.

[14] Liu Q , Li JF, Zhao L et al. Niobate-based lead-free piezoceramics: a diffused phase transition boundary leading to temperature-insensitive high piezoelectric voltage coefficients. J Mater Chem C 2018; 6: 1116–25.

[15] Wang D , Hussain F, Khesro A et al. Composition and temperature dependence of structure and piezoelectricity in (1−x)(K_1−y_Na_y_)NbO_3_-x (Bi_1/2_Na_1/2_)ZrO_3_ lead-free ceramics. J Am Ceram Soc 2017; 100: 627–37.

[16] Rubio-Marcos F , López-Juárez R, Rojas-Hernandez RE et al. Lead-free piezoceramics: revealing the role of the rhombohedral-tetragonal phase coexistence in enhancement of the piezoelectric properties. ACS Appl Mater Interfaces 2015; 7: 23080–8. 26436199 10.1021/acsami.5b06747

[17] Weyland F , Acosta M, Koruza J et al. Criticality: concept to enhance the piezoelectric and electrocaloric properties of ferroelectrics. Adv Funct Mater 2016; 26: 7326–33.

[18] Yao FZ , Wang K, Jo W et al. Diffused phase transition boosts thermal stability of high-performance lead-free piezoelectrics. Adv Funct Mater 2016; 26: 1217–24.

[19] Liu Q , Zhang Y, Zhao L et al. Simultaneous enhancement of piezoelectricity and temperature stability in (K,Na)NbO_3_-based lead-free piezoceramics by incorporating perovskite zirconates. J Mater Chem C 2018; 6: 10618–27.

[20] Damjanovic D . Comments on origins of enhanced piezoelectric properties in ferroelectrics. IEEE Trans Ultrason Ferroelectrics Freq Contr 2009; 56: 1574–85. 10.1109/TUFFC.2009.1222 19686973

[21] Li F , Zhang S, Xu Z et al. The contributions of polar nanoregions to the dielectric and piezoelectric responses in domain-engineered relaxor-PbTiO_3_ crystals. Adv Funct Mater 2017; 27: 1700310.

[22] Li F , Zhang S, Yang T et al. The origin of ultrahigh piezoelectricity in relaxor-ferroelectric solid solution crystals. Nat Commun 2016; 7: 13807. 27991504 10.1038/ncomms13807 PMC5187463

[23] Li F , Lin D, Chen Z et al. Ultrahigh piezoelectricity in ferroelectric ceramics by design. Nat Mater 2018; 17: 349–54. 29555999 10.1038/s41563-018-0034-4

[24] Nahas Y , Akbarzadeh A, Prokhorenko S et al. Microscopic origins of the large piezoelectricity of leadfree (Ba,Ca)(Zr,Ti)O_3_. Nat Commun 2017; 8: 15944. 28631724 10.1038/ncomms15944 PMC5481827

[25] Wu H , Zhang Y, Wu J et al. Microstructural origins of high piezoelectric performance: a pathway to practical lead-free materials. Adv Funct Mater 2019; 29: 1902911.

[26] Singh AK , Mishra SK, Ragini PD et al. Origin of high piezoelectric response of Pb(Zr_x_Ti_1-x_)O_3_ at the morphotropic phase boundary: role of elastic instability. Appl Phys Lett 2008; 92: 022910.

[27] Ahart M , Somayazulu M, Cohen R et al. Origin of morphotropic phase boundaries in ferroelectrics. Nature 2008; 451: 545–8. 18235495 10.1038/nature06459

[28] Hinterstein M , Hoelzel M, Rouquette J et al. Interplay of strain mechanisms in morphotropic piezoceramics. Acta Mater 2015; 94: 319–27.

[29] Hinterstein M , Rouquette J, Haines J et al. Structural description of the macroscopic piezo- and ferroelectric properties of lead zirconate titanate. Phys Rev Lett 2011; 107: 077602. 21902430 10.1103/PhysRevLett.107.077602

[30] Mgbemere HE , Hinterstein M, Schneider GA. Electrical and structural characterization of (K_x_Na_1-x_)NbO_3_ ceramics modified with Li and Ta. J Appl Crystallogr 2011; 44: 1080–9.

[31] Mgbemere H , Schneider G, Hoelzel M *et al*. Neutron diffraction study of (K_x_Na_1-x_)NbO_3_-based ceramics from low to high temperatures. J Appl Crystallogr 2016; 49: 891–901.

[32] Hinterstein M , Mgbemere H, Hoelzel M et al. Influence of microstructure on symmetry determination of piezoceramics. J Appl Crystallogr 2018; 51: 670–8.

[33] Saines PJ , Kennedy BJ, Elcombe MM. Structural phase transitions and crystal chemistry of the series Ba_2_LnB’O_6_ (Ln= lanthanide and B’= Nb^5+^ or Sb^5+^). J Solid State Chem 2007; 180: 401–9.

[34] Thomann H . A covalency model of ferroic phase transitions in perovskites. Ferroelectrics 1987; 73: 183–99.

[35] Shannon RD . Revised effective ionic radii and systematic studies of interatomic distances in halides and chalcogenides. Acta Crystallogr A 1976; 32: 751–67.

[36] Schütz D , Deluca M, Krauss W et al. Lone-pair-induced covalency as the cause of temperature- and field-induced instabilities in bismuth sodium titanate. Adv Funct Mater 2012; 22: 2285–94.

[37] Chang Y , Yang Z, Xiong L et al. Phase structure, microstructure, and electrical properties of Sb-modified (K,Na,Li)(Nb,Ta)O_3_ piezoelectric ceramics. J Am Ceram Soc 2008; 91: 2211–6.

[38] Tripathi R , Wood SM, Islam MS *et al*. Na-ion mobility in layered Na_2_FePO_4_F and olivine Na[Fe,Mn]PO_4_. Energy Environ Sci 2013; 6: 2257–64.

[39] Liu Q , Zhu FY, Zhao L et al. Further enhancing piezoelectric properties by adding MnO_2_ in AgSbO_3_-modified (Li,K,Na)(Nb,Ta)O_3_ lead-free piezoceramics. J Am Ceram Soc 2016; 99: 3670–6.

[40] Zhang MH , Wang K, Du YJ et al. High and temperature-insensitive piezoelectric strain in alkali niobate lead-free perovskite. J Am Chem Soc 2017; 139: 3889–95. 28233999 10.1021/jacs.7b00520

[41] Wang K , Yao FZ, Jo W et al. Temperature-insensitive (K,Na)NbO_3_-based lead-free piezoactuator ceramics. Adv Funct Mater 2013; 23: 4079–86.

[42] Qin Y , Zhang J, Yao W et al. Domain configuration and thermal stability of (K_0.48_Na_0.52_)(Nb_0.96_Sb_0.04_)O_3_-Bi_0.50_(Na_0.82_K_0.18_)_0.50_ZrO_3_ piezoceramics with high *d*_33_ coefficient. ACS Appl Mater Interfaces 2016; 8: 7257–65. 26942654 10.1021/acsami.6b00377

[43] Liu B , Li P, Shen B et al. Simultaneously enhanced piezoelectric response and piezoelectric voltage coefficient in textured KNN-based ceramics. J Am Ceram Soc 2018; 101: 265–73.

[44] Zhou JS , Wang K, Yao FZ et al. Multi-scale thermal stability of niobate-based lead-free piezoceramics with large piezoelectricity. J Mater Chem C 2015; 3: 8780–7.

[45] Liu Q , Zhang Y, Gao J et al. High-performance lead-free piezoelectrics with local structural heterogeneity. Energy Environ Sci 2019; 11: 3531–9.

[46] Wu H , Xue D, Lv D et al. Microstructure at morphotropic phase boundary in Pb(Mg_1/3_Nb_2/3_) O_3_-PbTiO_3_ ceramic: coexistence of nano-scaled {110}-type rhombohedral twin and {110}-type tetragonal twin. J Appl Phys 2012; 112: 052004.

[47] Theissmann R , Schmitt LA, Kling J et al. Nanodomains in morphotropic lead zirconate titanate ceramics: on the origin of the strong piezoelectric effect. J Appl Phys 2007; 102: 024111.

[48] Schmitt LA , Schönau KA, Theissmann R et al. Composition dependence of the domain configuration and size in Pb (Zr_1-x_Ti_x_) O_3_ ceramics. J Appl Phys 2007; 101: 074107.

[49] Schönau KA , Schmitt LA, Knapp M et al. Nanodomain structure of Pb (Zr_1-x_Ti_x_)O_3_ at its morphotropic phase boundary: investigations from local to average structure. Phys Rev B 2007; 75: 184117.

[50] Fu J , Zuo R, Xu Z. High piezoelectric activity in (Na,K)NbO_3_ based lead-free piezoelectric ceramics: contribution of nanodomains. Appl Phys Lett 2011; 99: 062901.

[51] Hu YH , Chan HM, Wen ZX *et al*. Scanning electron microscopy and transmission electron microscopy study of ferroelectric domains in doped BaTiO_3_. J Am Ceram Soc 1986; 69: 594–602.

[52] Tu CS , Hsieh CM, Chien R et al. Nanotwins and phases in high-strain Pb(Mg_1/3_Nb_2/3_)_1-x_Ti_x_O_3_ crystal. J Appl Phys 2008; 103: 074117.

[53] Liu W , Ren X. Large piezoelectric effect in Pb-free ceramics. Phys Rev Lett 2009; 103: 257602. 20366285 10.1103/PhysRevLett.103.257602

[54] Rabe KM , Ahn CH, Triscone JM. Physics of Ferroelectrics: A Modern Perspective. Berlin: Springer, 2007, 175–217.

[55] Damjanovic D . A morphotropic phase boundary system based on polarization rotation and polarization extension. Appl Phys Lett 2010; 97: 062906.

[56] Iwata M , Ishibashi Y. Phenomenological theory of morphotropic phase boundary with monoclinic phase in solid-solution systems of perovskite-type oxide ferroelectrics. Jpn J Appl Phys 2005; 44: 3095.

[57] Liu Q , Zhu FY, Zhang BP et al. Dielectric and ferroelectric properties of AgSbO_3_-modified (Li,K,Na)(Nb,Ta)O_3_ lead-free piezoceramics. J Mater Sci Mater Electron 2015; 26: 9309–15.

