# Supplementary material for: Practical high-performance lead-free piezoelectrics: structural flexibility beyond utilizing multiphase coexistence
Source: Natl Sci Rev. 2019 Nov 5;7(2):355–65. doi: 10.1093/nsr/nwz167 (PMC8288886; doi:10.1093/nsr/nwz167)
Supplement: nwz167_Supplemental_File [file nwz167_supplemental_file.doc]

Supporting Information

**Practical High-Performance Lead-Free Piezoelectrics : Structural Flexibility Beyond Utilizing Multiphase Coexistence**

*Qing Liu^1^, Yichi Zhang^1^*, Jing Gao^1^, Zhen Zhou^1^,Dong Yang^1^,Kai-Yang Lee^2^, Andrew Studer^3^, Manuel Hinterstein^2^, Ke Wang^1^, Xiaowen Zhang^1^, Longtu Li^1^, Jing-Feng Li^1*^*

^1^ State Key Laboratory of New Ceramics and Fine Processing, School of Materials Science and Engineering, Tsinghua University, Beijing 100084, China

^2^ Institute for Applied Materials (IAM), Karlsruhe Institute of Technology (KIT), 76131 Karlsruhe, Germany

^3^Australian Centre for Neutron Scattering, Australian Nuclear Science and Technology Organisation, Lucas Heights, New South Wales 2234, Australia

* Corresponding author:

jingfeng@mail.tsinghua.edu.cn ( Jing-Feng Li )

yichi-zhang@mail.tsinghua.edu.cn ( Yichi Zhang )

Keywords: Piezoelectricity; Lead-free; Potassium-sodium niobate; Structural flexibility; Temperature stability


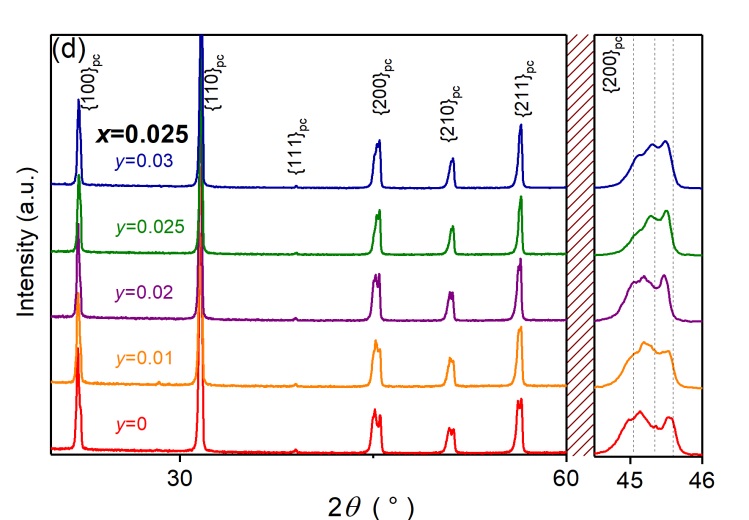

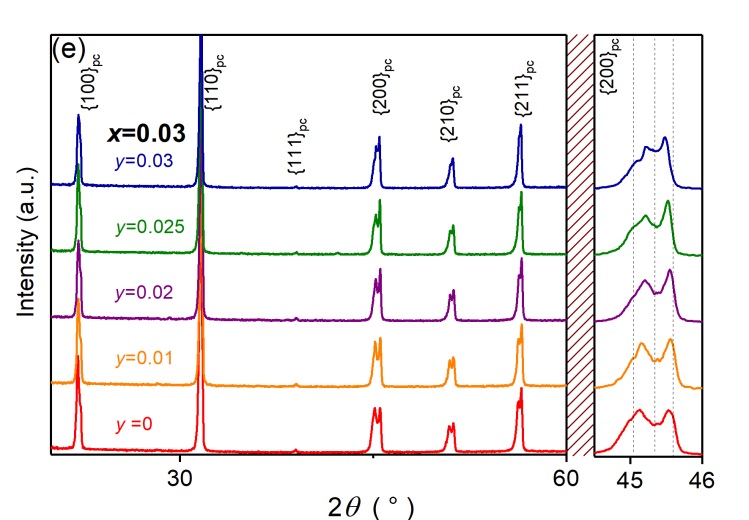

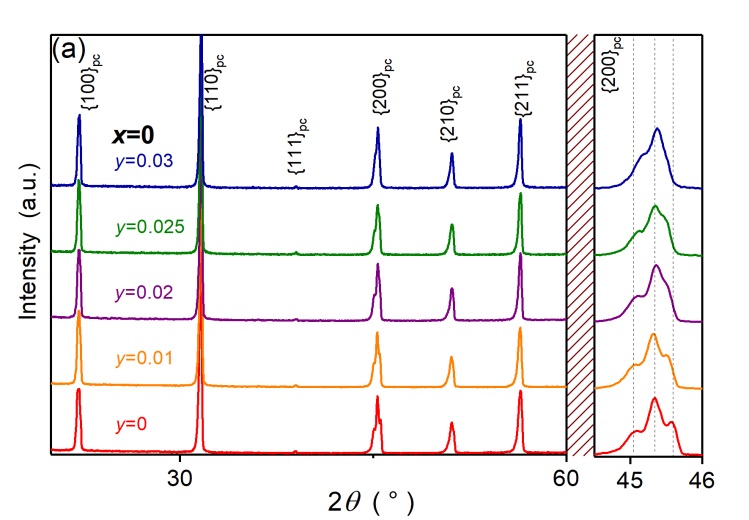

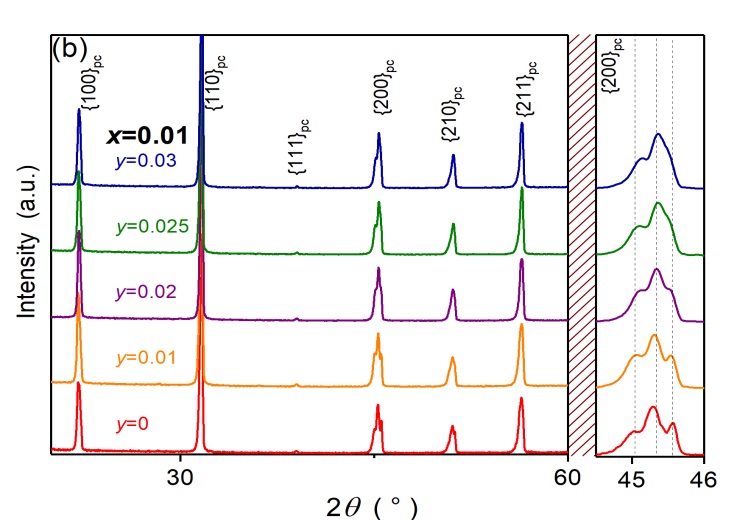

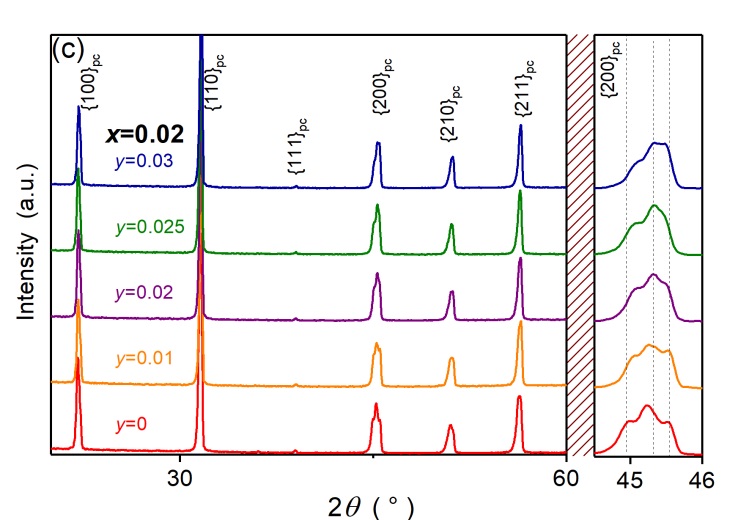

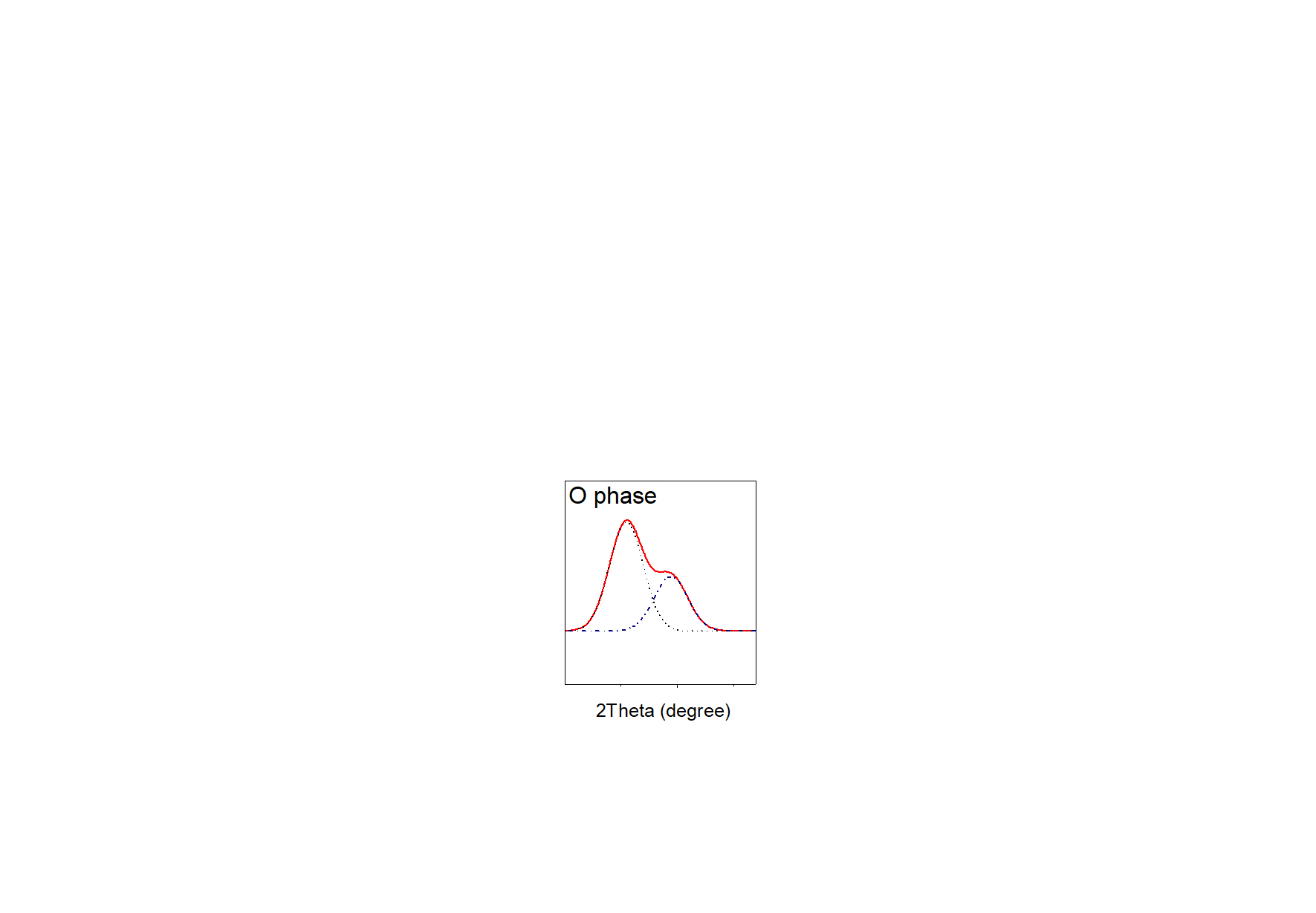

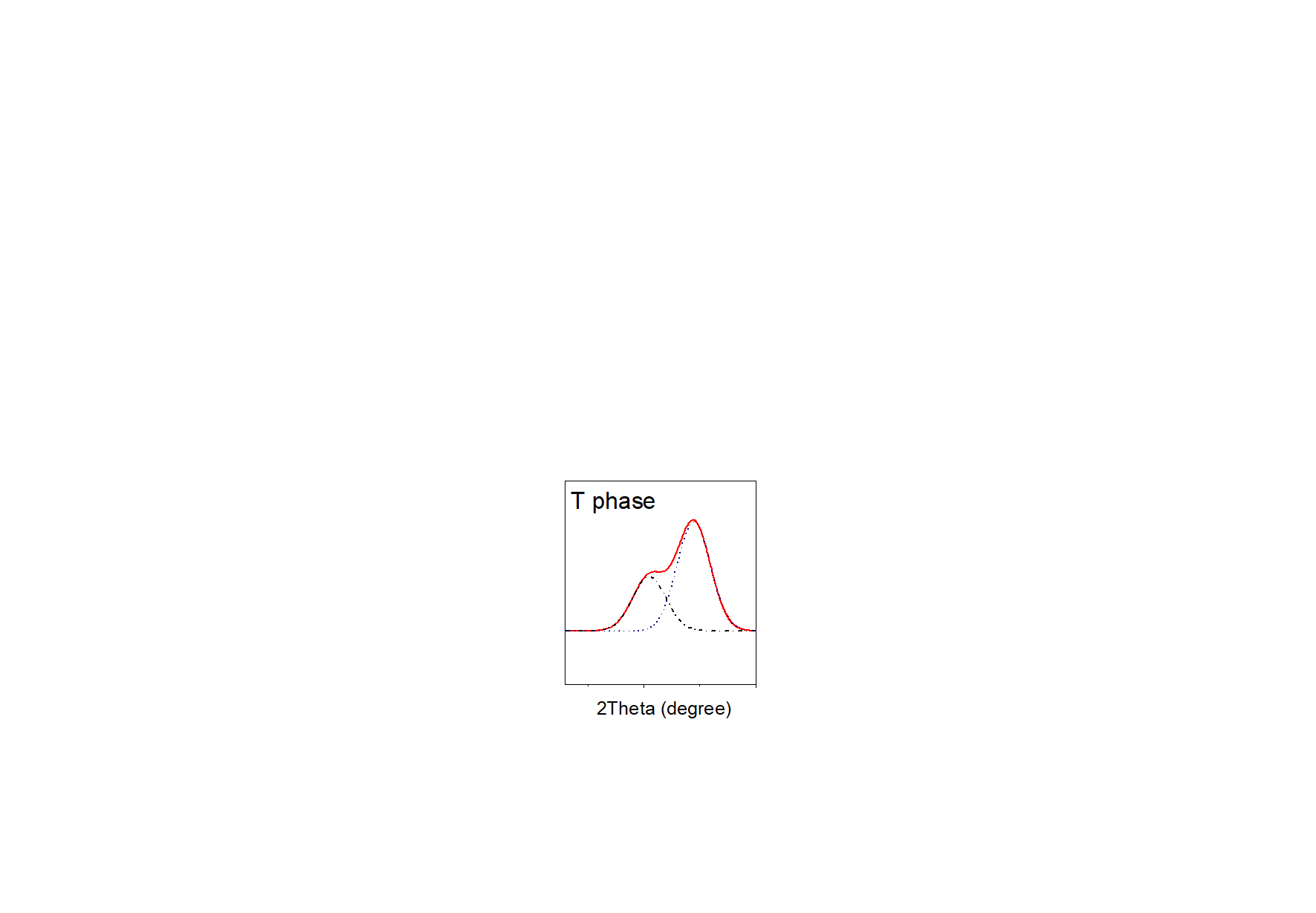

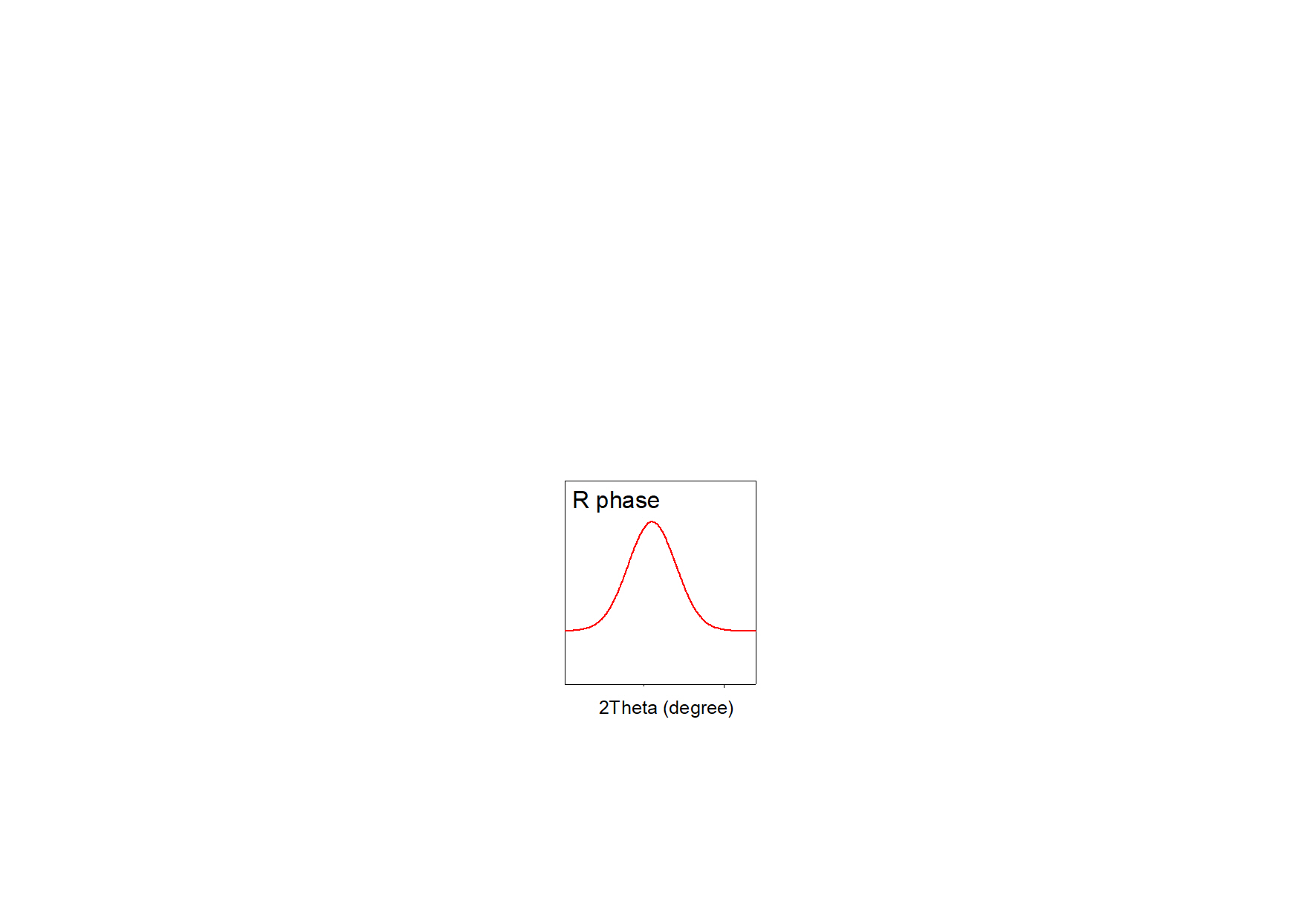


(f)

**Figure S1**. XRD patterns for the L_x_KNNS_y_-5BZ-2BNZ-1Mn samples.


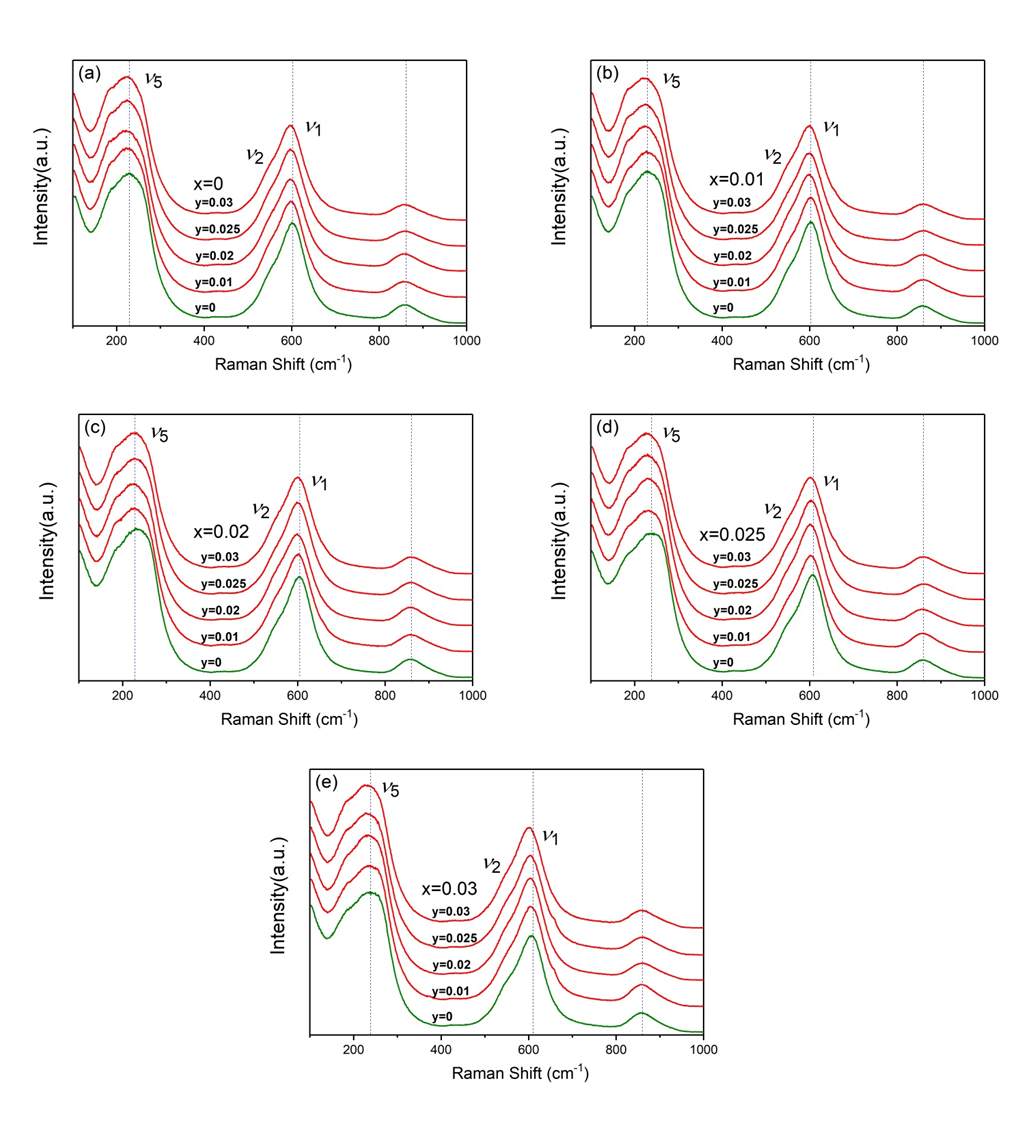


**Figure S1.(a)-(e)** Room-temperature XRD patterns of the L_x_KNNS_y_-5BZ-2BNH-1Mn samples. (f) The typical shapes of the 002_pc_ XRD reflection peaks around 2θ≈45° for the pure R, O and T phase, respectively.


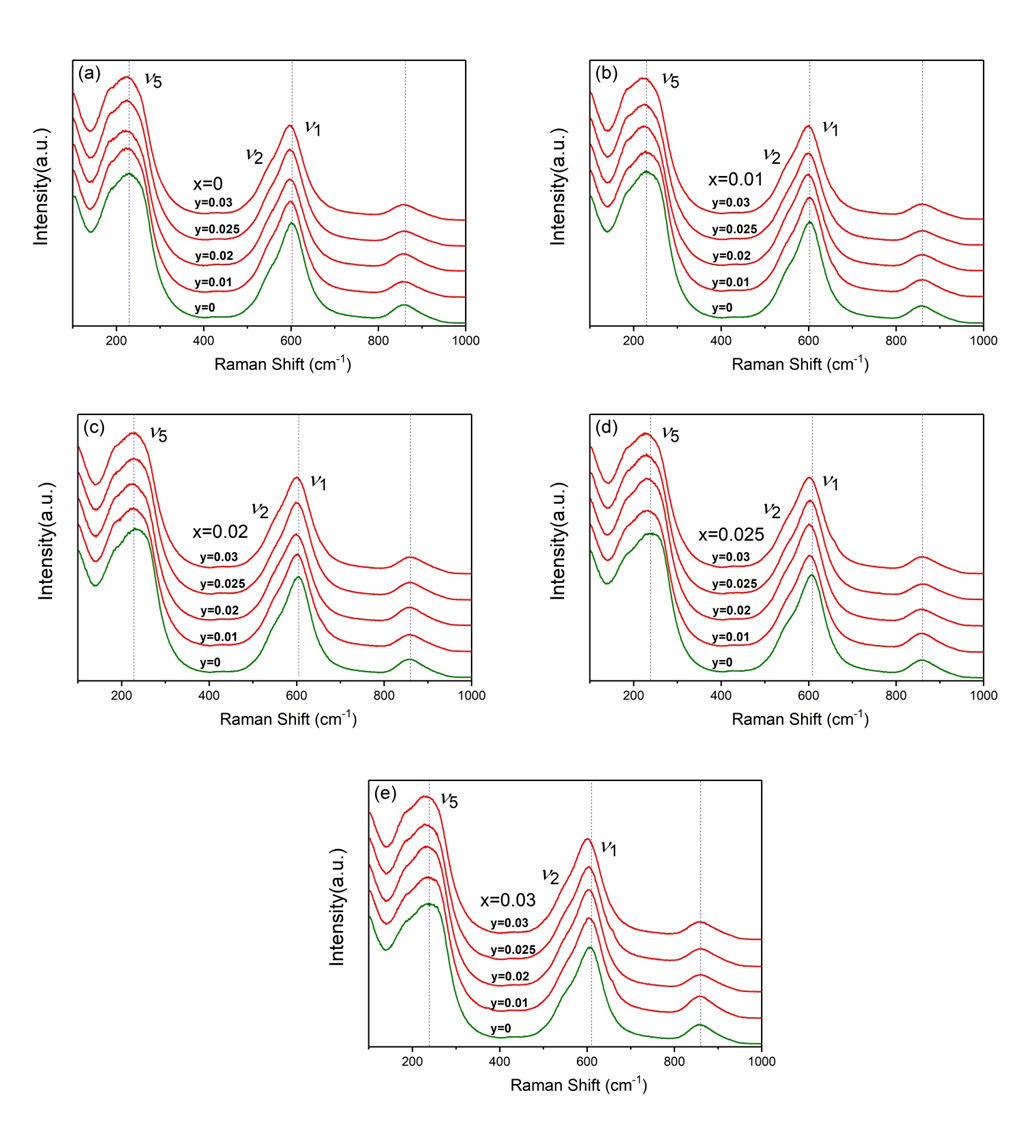


**Figure S2.(a)-(e)** Room-temperature Raman spectra of the L_x_KNNS_y_-5BZ-2BNH-1Mn samples.


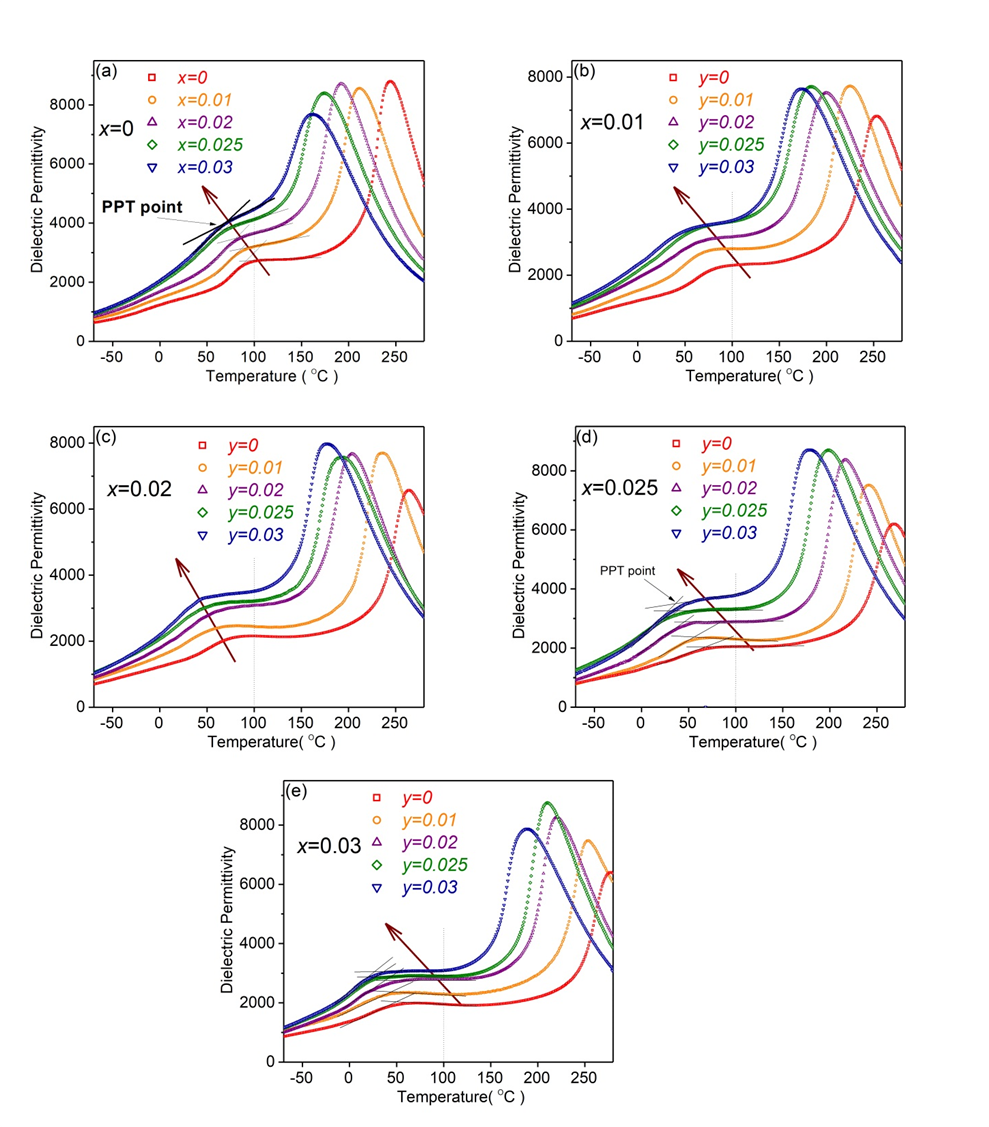


**Figure S3**. The temperature dependence of the dielectric permittivity for the L_x_KNNS_y_-5BZ-2BNZ-1Mn samples.


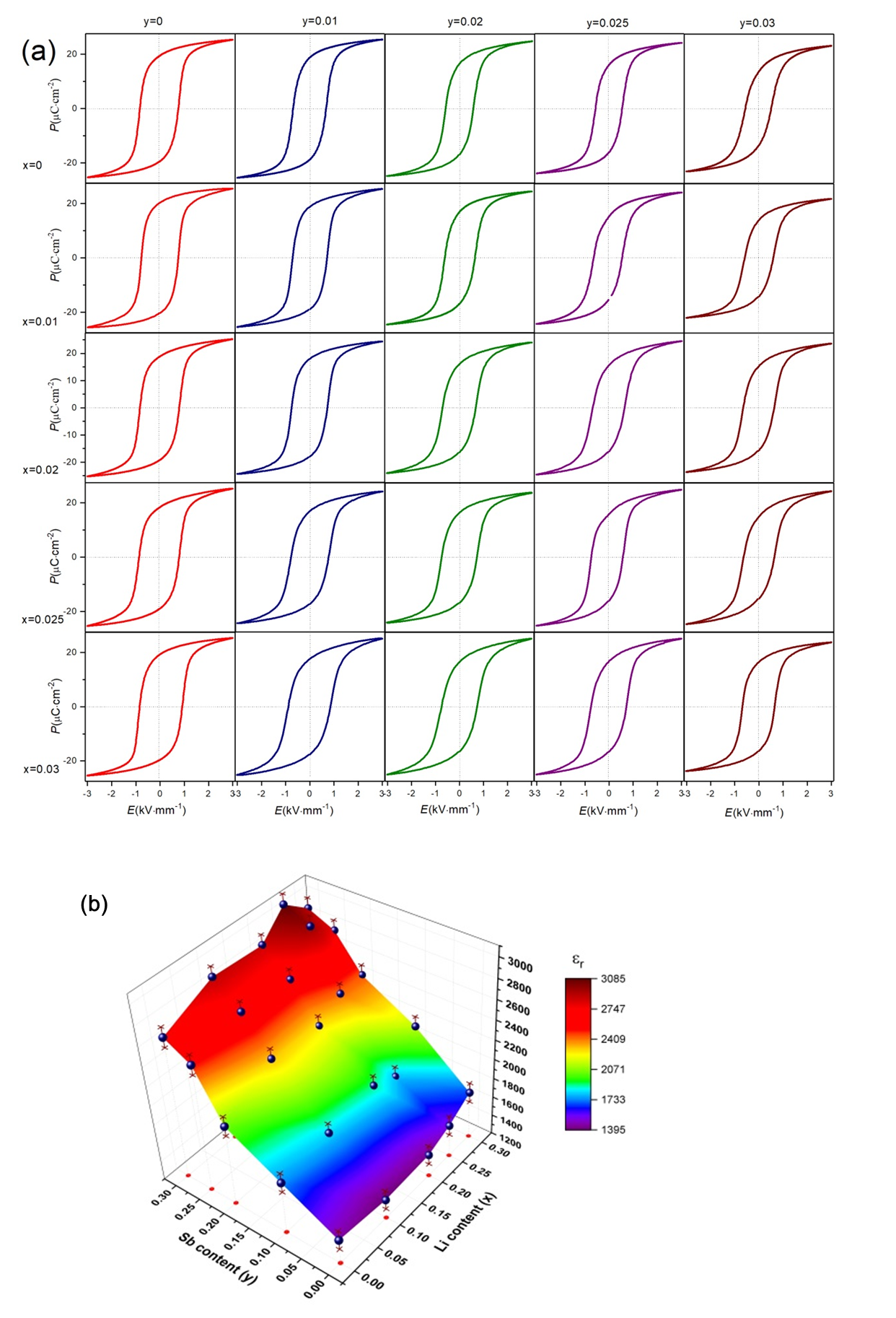


**Figure S4**. **(a)** The room-temperature *P-E* loops of the L*_x_*KNNS*_y_*-5BZ-2BNZ-1Mn samples. **(b)** The room-temperature dielectric coefficient *ε*_r_ of the L*_x_*KNNS*_y_*-5BZ-2BNZ-1Mn samples.

As shown in **Figure S4 (a)**, the P-E loop changed to be slimmer as Sb contents increased, indicating that a lower *E*_c_  could be obtained in the ceramic samples with higher Sb contents. According to these *P*-*E* loops, the room-temperature remanent polarization *P*_r_ of the L_x_KNNS_y_-5BZ-2BNZ samples is extracted as exhibited in **Figure 2d**. The decrease of *P*_r_ was only observed when increasing Sb contents. Li-modified ceramics exhibited a relatively larger remnant polarization. **Figure** **S4**(**b**) exhibits the room-temperature dielectric coefficient of the L_x_KNNS_y_-5BZ-2BNZ samples. The increase of the room-temperature dielectricity was observed when doping either Li or Sb element into the KNN matrix. It should be noted that Sb-modified ceramics demonstrated much larger dielectric coefficient than the Li-modified ceramics. In addition, Li can reduce the permittivity at other temperature as shown in **Figure** **S3**. The Li-induced enhancement of dielectricity should be mainly attributed to the fact that the phase transition point approaches room temperature as indicated by the temperature dependence of dielectric permittivity.

Sb is endowed with the ability to induce more relaxor characteristic in the KNN-based ceramics and increase the local inhomogeneity while Li can benefit the sintering of the KNN-based ceramics, making the ceramics more homogeneous.^[1-3]^ With a smaller radius, Li is considered to have an ability to elevate the degree of the cationic ordering.^[4]^ Increasing Sb contents can hugely enhance the dielectric property due to the softening of the lattice associated with the reduced unit cell distortion and the more covalent characteristic of KNN matrix.^[5-9]^ The onset of cationic disorder is unavoidable in this complex system, the magnitude of which can hugely affect the piezoelectric performance since the long-range ordered ferroelectric structure relies on the cationic distribution. In addition to the fabrication processing, dopants and their contents can influence the cationic distribution, resulting in the local structural heterogeneity. The possible nanoscale structural heterogeneity produced by Sb doping can also destabilize the long-range ordering of dipoles, which contributes to the enhancement of the dielectric property.^[10]^ However, the significant loss of macroscopic polarization occurs when the doping of Sb is excessive. The decreased unit cell distortion can give rise to the reduction of the spontaneous polarization and then leads to the reduction of the macroscopic remanent polarization. It should be noted that the loss of macroscopic polarization should be mainly attributed to the severe disruption of the long-range ferroelectric order due to increased degree of the local inhomogeneity. This can be corroborated by the fact that other composition further modified by Li with a similar unit cell distortion can also exhibit a much larger macroscopic remanent polarization as shown in **Figure 2d**. The enhancement of permittivity and the maintaining of macroscopic remanent polarization were achieved by introducing both Sb and Li elements into the ceramics.


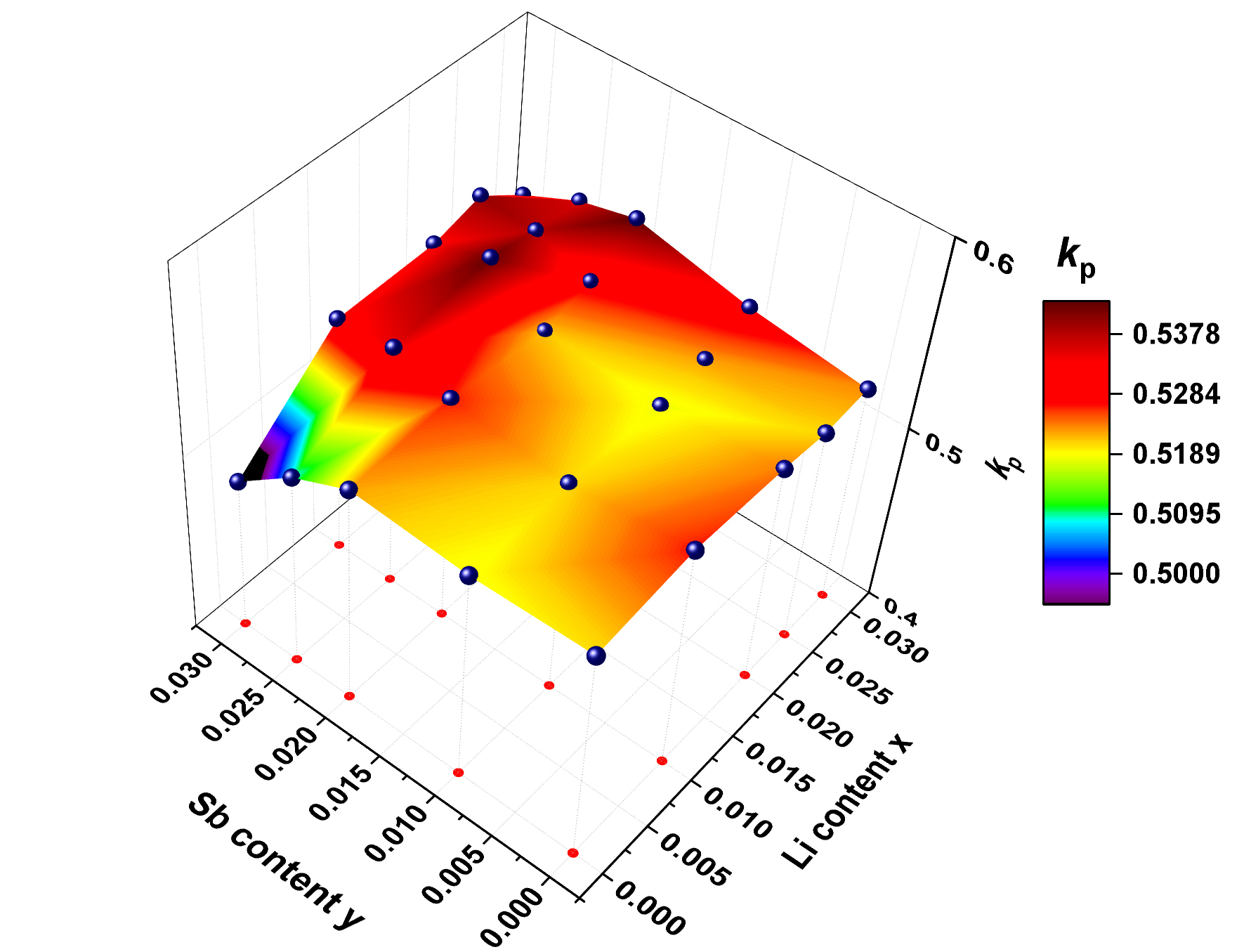


**Figure S5**. The room-temperature planar electromechanical coupling factor *k*_p_ of the L*_x_*KNNS*_y_*-5BZ-2BNZ-1Mn samples.


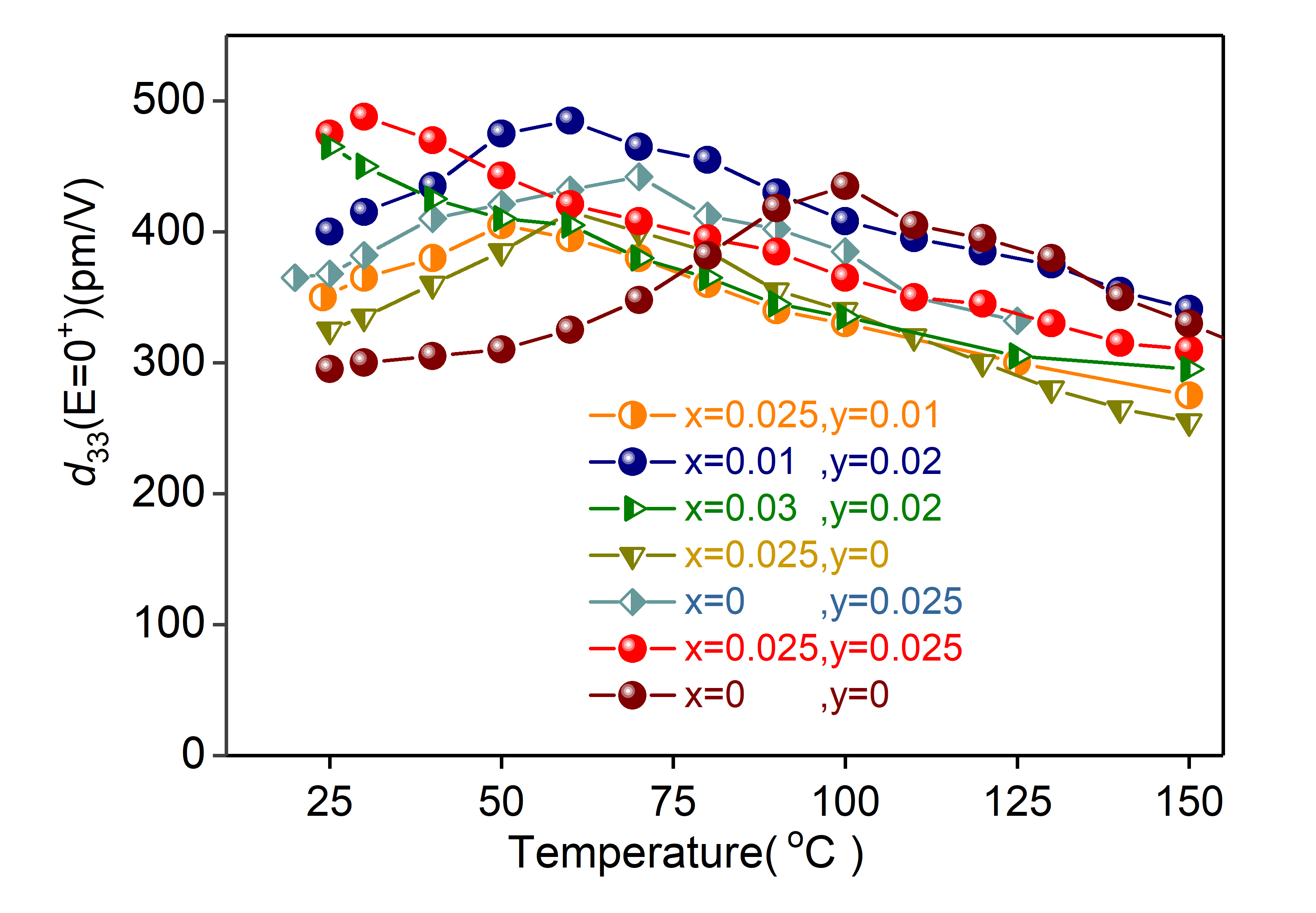


**Figure S6**. The temperature dependence of the *d*_33_(E=0^+^) for the representative L*_x_*KNNS*_y_*-5BZ-2BNH-1Mn ceramics.


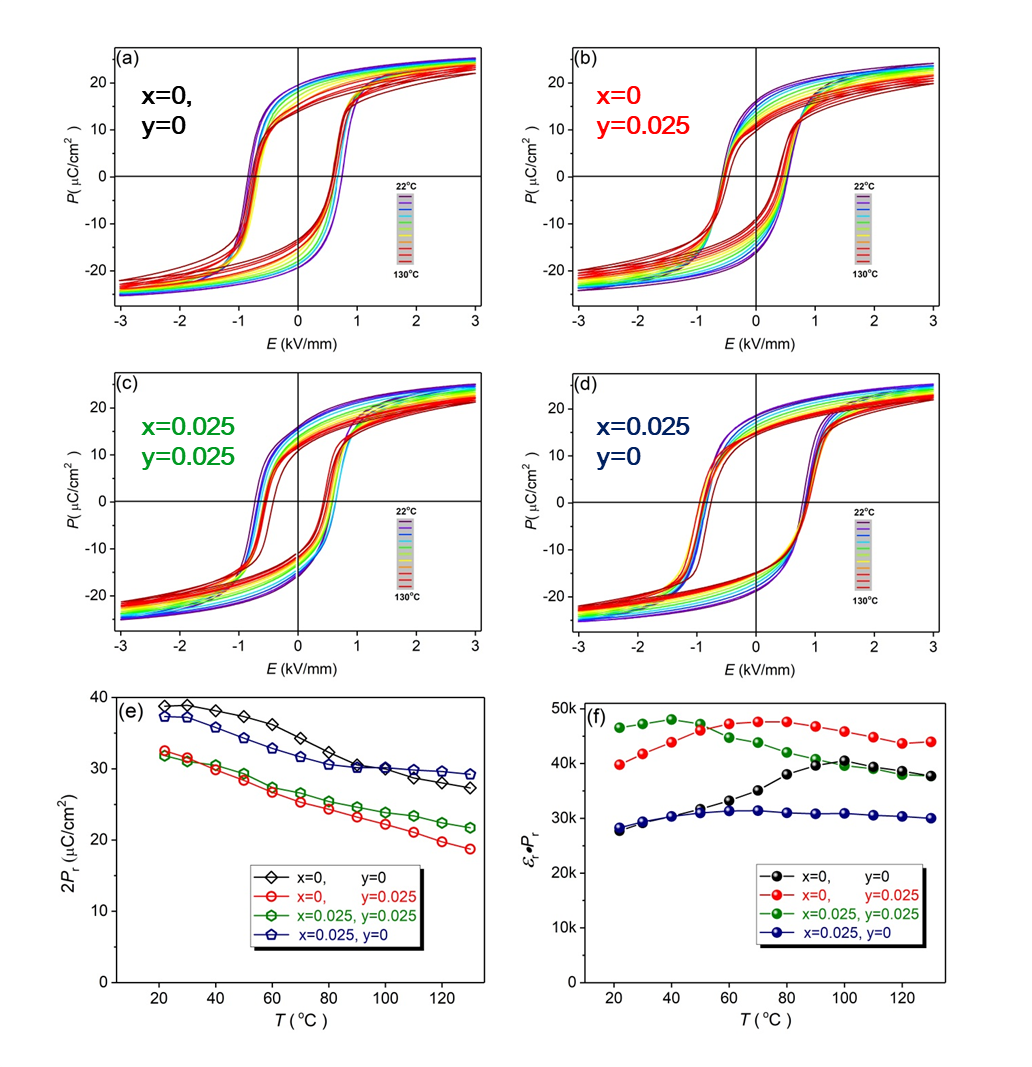


**Figure S7.** The P-E hysteresis loops for the *x*=0, *y*=0 (a), *x*=0, *y*=0.025 (b), *x*=0.025, *y*=0.025 (c), *x*=0.025, *y*=0 (d) samples.(e) Temperature dependence of 2*P_r_* for the representative L_x_KNNS_y_-5BZ-2BNH-1Mn ceramics.(f) Temperature dependence of *ε_r_*·*P_r_* value for the representative L_x_KNNS_y_-5BZ-2BNH-1Mn ceramics.

*P_r_* monotonically decreased during the temperature span of phase transition between two competing ferroelectric symmetries as exemplified in **Figure S7(e)** while *ε*_r_ firstly increased and then decreased as exemplified in **Figure S3**. It is worth nothing that the high-temperature ferroelectric phase (tetragonal phase) exhibited a much larger *ε*_r_ *P_r_* than low-temperature ferroelectric phase (orthorhombic or rhombohedral phase) due to the fact that the growth of the *ε*_r_ was much larger than the deterioration of the *P_r_*. For a composition, the largest *ε*_r_*P_r_* generally appears around the phase transition point owing to dielectric abnormity. Compared with the single tetragonal phase, the enhancement of the *d*_33_ (E=0^+^) or *ε*_r_*P_r_* due to the piezoelectric anomaly induced by the PPT effect might only constitute approximately 25% of the whole *d_33_ (E=0^+^)* or *ε_r_P_r_* value while the vast majority of piezoelectric response is considered to rely on the softening of the structure tailored by the various elements. Thus, the Sb induced lattice softening rather than the PPT effect was considered to be the main source of the dielectric enhancement when comparing the permittivity around phase transition temperature with that of the single tetragonal phase.


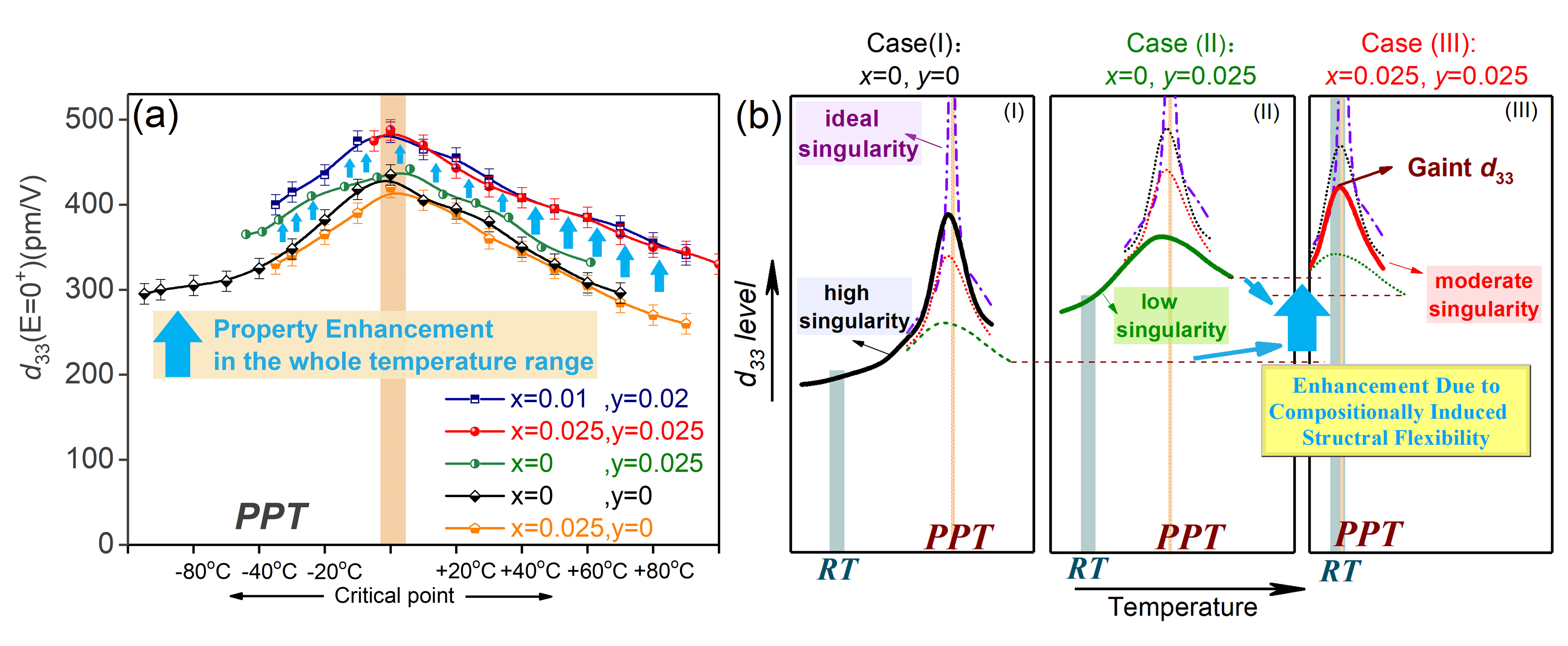


**Figure S8.** (a) The variations of the *d*_33_ (E=0^+^) of the representative L_x_KNNS_y_-5BZ-2BNH-1Mn ceramics, which are centered according to the temperature of the peak *d*_33_ ^*^(E=0) value for easy comparison. (b) The sketched diagram elaborating on the composition-induced room-temperature *d*_33_ change associated with singularity of the critical behavior, phase transition point and compositionally induced structural flexibility associated with lattice softening and reduced unit cell distortion.

To give insight into the possible origins for piezoelectricity enhancement apart from the PPT effects (temperature), the variation of the *d*_33_(E=0^+^) of the representative L_x_KNNS_y_-5BZ-2BNH-1Mn ceramics are centered according to the temperature of the peak *d*_33_ (E=0) values (or critical point/ phase transition point) for the ease of comparison as shown in **Figure S8(a)**. The *d*_33_(E=0^+^) demonstrated an arch-shaped variation around the phase transition point. It is obvious that Sb-modified samples showed an increase of the *d*_33_ (E=0^+^) level in the whole-temperature range while doping Li did not enhance but slightly decreased *d*_33_. Therefore, this enhancement could not be entirely attributed to the temperature-dependent PPT effects but also be associated with composition-induced structural instability. According to the above investigations, a sketched diagram was proposed to illustrate the room-temperature high piezoelectricity originated from the critical behavior and structural flexibility associated with lattice softening^[1, 2]^ and reduced unit cell distortion in the Li and Sb co-modified ceramics as shown in **Figure S8(b).** According to the Ginzburg–Landau model, the general relationship between *d*_33_, electrostrictive coefficient *Q*_11_, dielectric susceptibility χ_33_, polarization *P*_3_ and the Gibbs free energy *G* can be derived as *d*_33_=2*ε*_33_*Q*_11_*P*_3_=2*ε*_0_*χ*_33_*Q*_11_*P*_3_=2*ε*_0_ *1/( ∂^2^G/∂P_3_^2^)Q*_11_*P*_3_.^[3, 4]^ Due to the symmetry breaking, the dielectric susceptibility diverges at the transition.^[3]^ Thus, the property enhancement from polymorphous phase transition (PPT) effect is essentially a consequence of the temperature-induced flattening of the Gibbs free energy profile and can be viewed as a critical phenomenon.^[4-7]^ In single crystals, due to the sharp change of the free energy, the electrical properties are highly sensitive to temperature at PPT ^[8]^, which is supported by both *ab initio* and phenomenological theoretical studies.^[9-13]^ In ceramics, due to the existence of the local heterogeneities and the disappearance of macroscopic orientation as well as the intergranular stress relaxation, the sharply defined phase transition temperature point can change into a broadened temperature range and the temperature sensitivity of the electric properties is milder compared to the single crystal.^[7]^ Owing to the unavoidable disorders and impurities in an actual real-world sample, the phase transition appears more diffuse, resulting in finite derivatives of various parameters affected by symmetry change at the transition point. The disorders can affect the critical exponents and significantly weaken the singularity, giving rise to the decreased amplitude of the finite maxima.^[14]^ Thus, larger peak piezoelectricity due to the PPT effect around the phase transition point is expected in the ceramics with a sharper phase transition behavior, which might result from an ordered and homogenous structure.^[15, 16]^ Doping Sb can shift the temperature span of piezoelectricity anomaly toward room temperature and achieve the enhancement of the piezoelectricity in the whole temperature range via introducing structural instability associated with lattice softening and reduced unit cell distortion. However, in this work, the slightly degraded singularity also occurs due to the Sb-induced heterogeneity.^[17]^ The appropriate amount of Li could benefit the sintering of the KNN-based ceramics, contributing to the homogeneity. With a small radius, Li is also considered to have the ability to elevate the degree of the cationic ordering. ^[18]^ Thus, the large piezoelectricity in the composition *x*=0.025, *y*=0.025 results from the piezoelectricity anomaly with moderate singularity due to the polymorph phase transition and the temperature-independent but compositional-induced piezoelectricity enhancement, which originated from structural flexibility associated with lattice softening and reduced unit cell distortion.


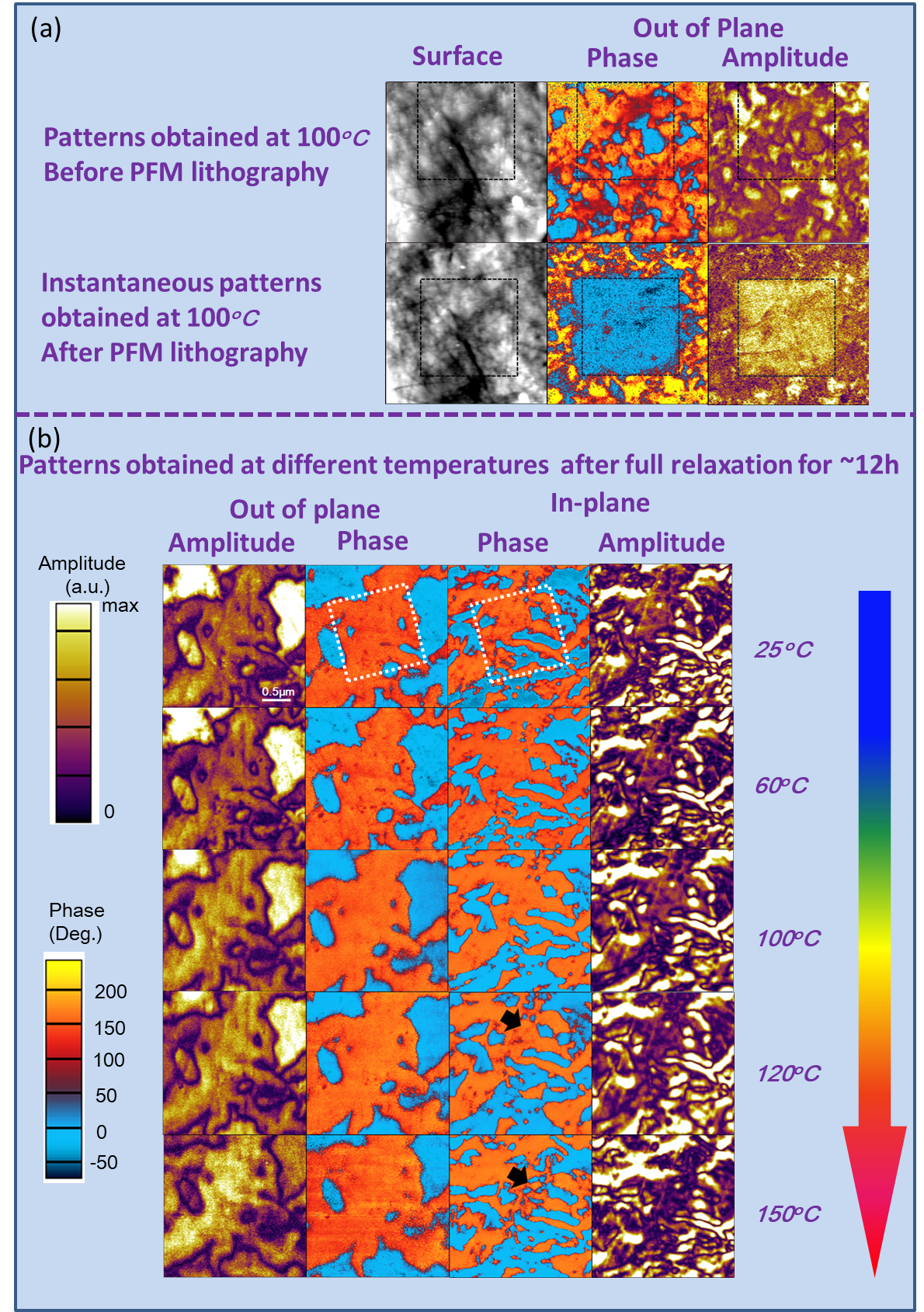


**Figure S9.** (a) Local poling experiments conducted on the *x*=0.025, *y*=0.025 sample within a square region of side length 5 um at 100 ºC with an electric field 30V. (b) The in-situ observation of the PFM images for the *x*=0.025, *y*=0.025 sample at different temperatures, which was conducted after the sample in the local poling state had been fully relaxed for 12h.

Local poling experiments were first conducted on the sample with *x*=0.025, *y*=0.025 in a square region of side length 5 um at 100 ºC with an electric field 30V. After full relaxation for 12h, the in-situ observation of domain morphology was conducted at different temperatures as shown in **Figure S9**. For both lateral piezoresponse force microscope (LPFM) images and vector piezoresponse force microscope (VFPM) images were in accord with its corresponding amplitude images. The LPFM image exhibited more complicate domain patterns than the VFPM image because the local poling conducted in the vertical direction played a less impact on the virgin lateral orientation of domains. Besides, furthermore, the domain patterns of the unpoled regions also changed in VPFM images after a long time relaxation, which could be attributed to the elastic tensor to the local stress induced by the local poling. As temperature increased from 25C to 110 ºC, no significant change could be observed both in VPFM and LPFM images. With further elevating the temperature to 150 ºC , most part of the LPFM domain morphology was maintained but some small irregular island-shaped domain patterns occurred within the bigger region exampled by the black arrow, indicating a more severe domain reorientation in the lateral direction than vertical direction.


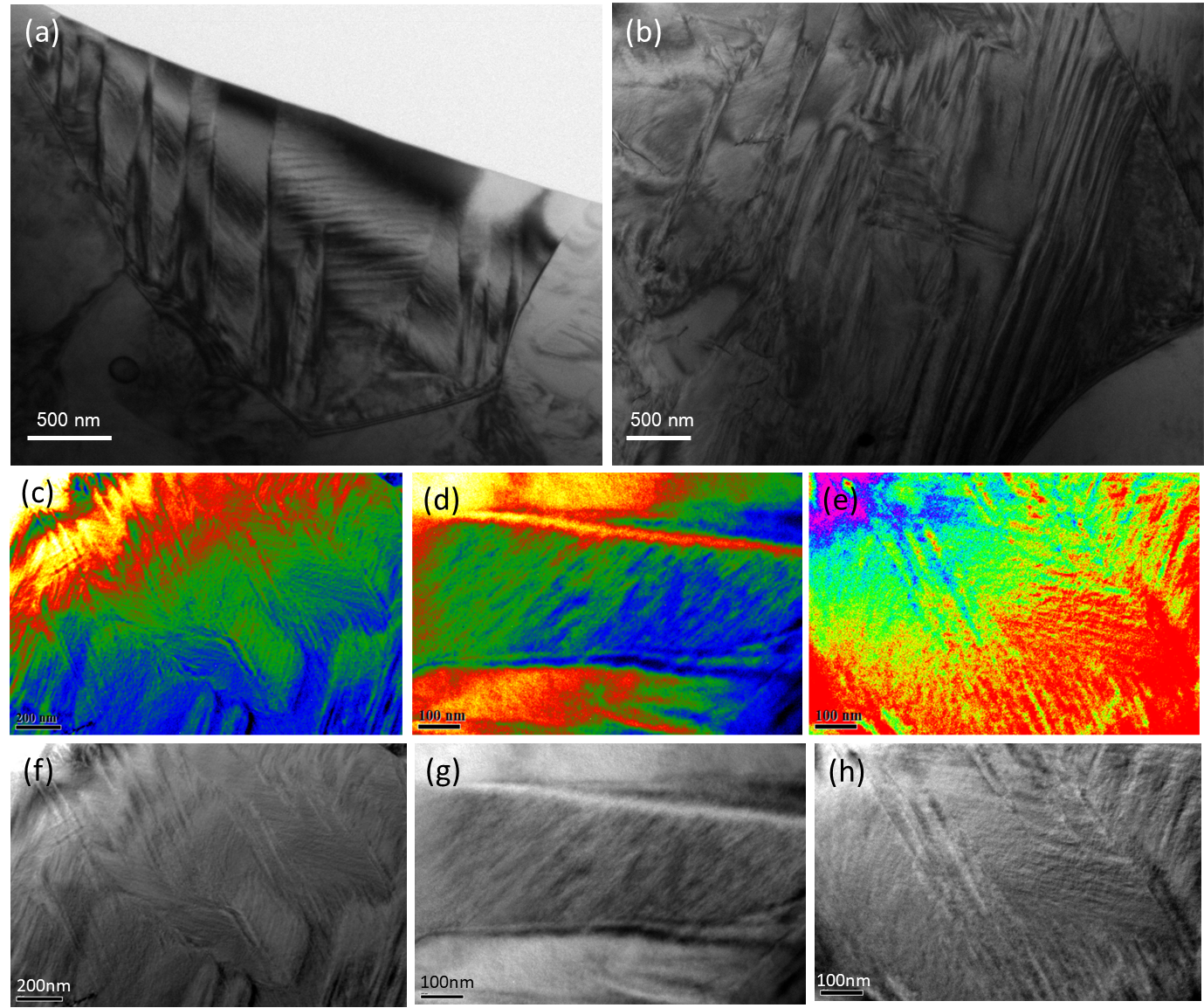


**Figure S10**. Bright-field TEM images of the *x*=0.025, *y*=0.025 sample showing strip-like and fibrous patterns as well as hierarchical nanodomains.

References

[1] W. Liu, X. Ren, *Phy. Rev. Lett.* **2009**, *103*, 257602.

[2] H. Wu, D. Xue, D. Lv, J. Gao, S. Guo, Y. Zhou, X. Ding, C. Zhou, S. Yang, Y. Yang, *J. Appl. Phy.* **2012**, *112*, 052004.

[3] K. M. Rabe, C. H. Ahn, J.-M. Triscone, *Physics of Ferroelectrics: a Modern Perspective*, Springer Science & Business Media, New York, USA **2007**.

[4] D. Damjanovic, *IEEE Transactions on Ultrasonics Ferroelectrics and Frequency Control* **2009**, 56, 1574.

[5] Q. Liu, J.-F. Li, L. Zhao, Y. Zhang, J. Gao, W. Sun, K. Wang, L. Li, *J. Mater. Chem. C* **2018**, *6*, 1116.

[6] Q. Liu, Y. Zhang, L. Zhao, J. Gao, Z. Zhou, K. Wang, X. Zhang, L. Li, J.-F. Li, *J. Mater. Chem. C* **2018**, *6*, 10618.

[7] F. Weyland, M. Acosta, J. Koruza, P. Breckner, J. Rödel, N. Novak, *Adv. Funct. Mater.* **2016**, *26*, 7326.

[8] H. Liu, P. Veber, J. Rödel, D. Rytz, P. B. Fabritchnyi, M. I. Afanasov, E. A. Patterson, T. Frömling, M. Maglione, J. Koruza, *Acta Mater.* **2018**, *148*, 499.

[9] W. Duan, Z.-R. Liu, *Curr. Opin. Solid State Mater. Sci.* **2006**, *10*, 40.

[10] N. Huang, Z. Liu, Z. Wu, J. Wu, W. Duan, B.-L. Gu, X.-W. Zhang, *Phy. Rev. Lett.* **2003**, *91*, 067602.

[11] Z. Wu, R. E. Cohen, *Phy. Rev. Lett.* **2005**, *95*, 037601.

[12]A. Bell, *J Appl.Phy.* **2001**, *89*, 3907.

[13]M. Iwata, Y. Ishibashi, *Jpn. J Appl. Phy.* **2005**, *44*, 3095.

[14] B. V. Balagurov, V. Vaks, *Zh. Eksp. Teor. Fiz* **1973**, *65*, 1600.

[15] N. Setter, L. Cross, *J Appl.Phy.* **1980**, 51, 4356.

[16] C.-S. Hong, S.-Y. Chu, C.-C. Tsai, C.-C. Hsu, *Ceram. Int.* **2012**, *38*, 4123.

[17] Q. Liu, Y. Zhang, J. Gao, Z. Zhou, H. Wang, K. Wang, X. Zhang, L. Li, J.-F. Li, *Energ. Environ. Sci.* **2018**, *11*, 3531.

[18] W. Zhong, *Physics of Ferroelectricity*, Science Press*,* Beijing, China **1996**.
